# Cilia Control Vascular Mural Cell Recruitment in Vertebrates

**DOI:** 10.1016/j.celrep.2016.12.044

**Published:** 2017-01-24

**Authors:** Xiaowen Chen, Dafne Gays, Carlo Milia, Massimo M. Santoro

**Affiliations:** 1 Vesalius Research Center, VIB-KUL, Leuven 3000, Belgium; 2 Department of Molecular Biotechnology and Health Sciences, Molecular Biotechnology Center, University of Turin, Turin 10126, Italy

**Keywords:** blood flow, cilia, mural cells, CRISPR-Cas9, zebrafish model

## Abstract

Vascular mural cells (vMCs) are essential components of the vertebrate vascular system, controlling blood vessel maturation and homeostasis. Discrete molecular mechanisms have been associated with vMC development and differentiation. The function of hemodynamic forces in controlling vMC recruitment is unclear. Using transgenic lines marking developing vMCs in zebrafish embryos, we find that vMCs are recruited by arterial-fated vessels and that the process is flow dependent. We take advantage of tissue-specific CRISPR gene targeting to demonstrate that hemodynamic-dependent Notch activation and the ensuing arterial genetic program is driven by endothelial primary cilia. We also identify zebrafish *foxc1b* as a cilia-dependent Notch-specific target that is required within endothelial cells to drive vMC recruitment. In summary, we have identified a hemodynamic-dependent mechanism in the developing vasculature that controls vMC recruitment.

## Highlights

• Blood flow and cilia are required for vascular mural cell (vMC) coverage in zebrafish
• Cilia-dependent Notch signaling drives vMC recruitment of arterial-fated vessels
• Foxc1b is necessary and sufficient to drive vascular myogenesis in zebrafish

## Graphical Abstract

**Figure:**
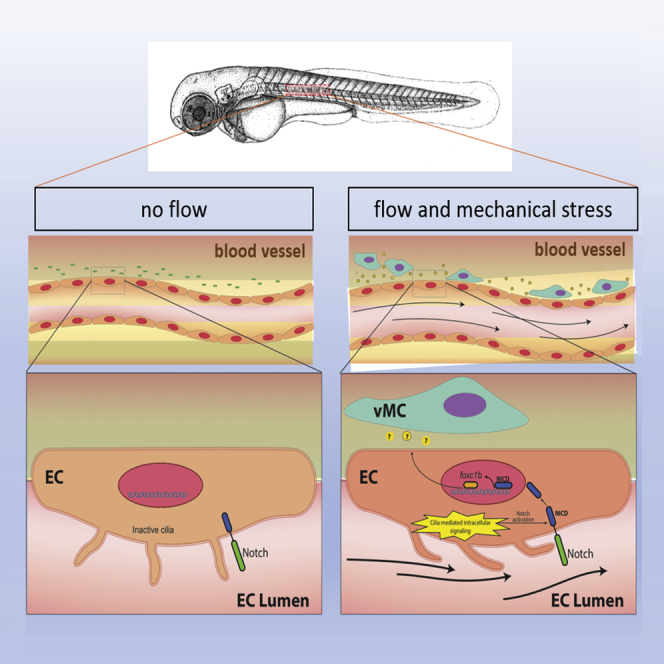

## Teaser

Chen et al. find that primary cilia are responsible for blood-flow-driven Notch activation in arterial vessels of zebrafish embryos. This pathway leads to specific expression of *foxc1b*, which then drives vMC recruitment and supports vascular myogenesis in zebrafish.

## Introduction

Vascular mural cells (vMCs) are an essential component of the vascular system and control cardiovascular development and maturation (Gaengel et al., 2009, van Dijk et al., 2015). In vertebrates, vMC coverage of the developing vasculature occurs when mesodermal precursors surround blood vessels and start to differentiate into vascular mural cells, a process also known as vascular myogenesis (Owens et al., 2004, Santoro et al., 2009, Wang et al., 2015, Yoshida and Owens, 2005). The origin, number, type, and organization of vMCs depend on the localization of the vessel and its function (Johnson, 2014, Majesky, 2007, Pfaltzgraff and Bader, 2015). Alterations in mural cell coverage or in its stable attachment to the endothelium is associated with several diseases, including diabetic retinopathy, venous malformation, and hereditary stroke (Armulik et al., 2011). Poor recruitment of mural cells in tumor vasculature is also implicated in the regulation of tumor growth and has thus been suggested as a potential anti-angiogenic target in cancer therapy as well as a hallmark of vascular normalization in tumors (Carmeliet and Jain, 2011).

Vascular mural cell (MC) recruitment and differentiation are related to the artero-venous identity of blood vessels. During early vascular development, specific mechanisms are responsible for the acquisition and retention of either arterial or venous identity (Simons and Eichmann, 2015). Zebrafish studies have helped to identify Notch and vascular endothelial growth factor (VEGF) signaling as critical molecular players involved in this process (Quillien et al., 2014, Siekmann and Lawson, 2007b). After the initial establishment of molecular differences between arterial and venous endothelial cells (ECs), other factors play different roles to further promote arteriogenesis and vascular maturation. One of them is blood flow, a critical biological regulator of vasculogenesis and vascular remodeling that initiates and supports multiple processes during vascular development and homeostasis (Boselli et al., 2015, Buschmann et al., 2010, Udan et al., 2013). As blood flows, the vascular wall is constantly subjected to physical forces, known as shear or mechano-stress, which are known to regulate important physiological blood vessel responses and are implicated in the development of arterial wall pathologies (Hahn and Schwartz, 2009). Several studies have clearly demonstrated that blood flow also contributes to arterial-venous specification and differentiation (le Noble et al., 2004). Indeed, even if genetic programs allow molecular distinction between arteries and veins before the onset of flow, there are no structural differences between these vessels at this stage. Although blood flow is thought to be responsible for the functional differentiation of arteries and veins, the role of hemodynamic forces and shear stress on MC recruitment around developing vasculature has been poorly investigated.

Primary cilia are sensory organelles that extend from the cell surface and sense extracellular signals (Goetz and Anderson, 2010). Primary cilia are necessary for shear stress sensing in different developing organs, such as the kidneys and blood vessels. Endothelial primary cilia protruding from the inner surface of blood vessel walls sense changes by blood-flow-dependent shear stress and convert this mechano-sensation into an intracellular molecular signal, triggering different cellular responses (Ando and Yamamoto, 2013, Hierck et al., 2008). It has been shown that endothelial primary cilia bend in response to blood flow forces and are necessary for flow sensing as well as the control of angiogenesis in normal and pathological conditions (Dinsmore and Reiter, 2016, Garcia-Gonzalez et al., 2010, Goetz et al., 2014, Kallakuri et al., 2015). Much less is known about whether cilia regulate vascular myogenesis and endothelial-specific pathways, such as Notch signaling.

In this work, we report a mechanism responsible for vMC recruitment in developing zebrafish vasculature. Using pharmacological and genetic approaches, we demonstrate that hemodynamic forces and cilial mechano-sensors are necessary to promote vascular myogenesis by activating arterial Notch signaling. We also show that this blood-flow-dependent Notch signaling leads to *foxc1b* expression in arteries, *foxc1b* being necessary and sufficient to drive vMC recruitment and differentiation, even in the absence of flow during vertebrate development.

## Results

### Zebrafish Vascular MCs Are Recruited Only by Arterial-Fated Vessels of Developing Vasculature

**Figure 1:**
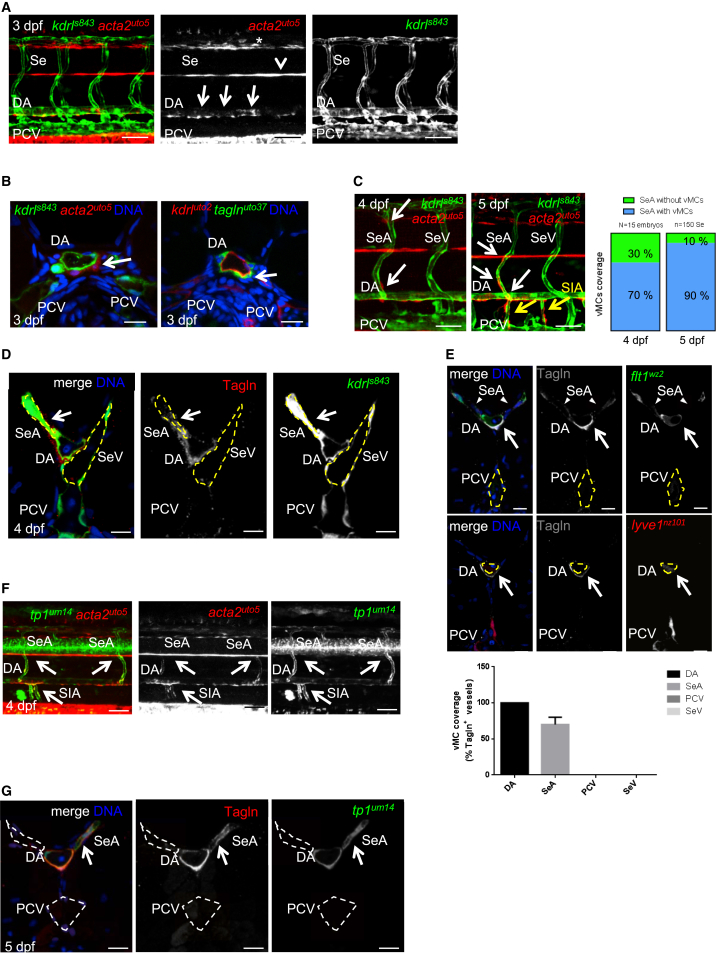
Zebrafish vMCs Are Recruited around Arterial-Fated Vessels of Developing Vasculature (A) DA, but not PCV, is covered by vMCs (red, arrow). Partial z-projection of the trunk region (somite 8–14) of a *Tg(kdrl:egfp)*^*s843*^; *Tg(acta2:mCherry)*^*uto5*^ embryo at 3 dpf. Merged and single channels are shown. *mCherry* expression is also detected in the lateral line (arrowhead) and floor plate (star). Scale bar, 100 μm. (B) Confocal transverse sections of the DA of Tg embryos of the indicated genotype. Both *Tg(acta2:mCherry)*^*uto5*^ and *Tg(tagln:CAAX-egfp)*^*uto37*^ lines mark vMCs located in the ventral side of the DA (arrow). Blue, nuclei. Scale bar, 25 μm. (C) vMCs are recruited only by segmental arteries. At 4 and 5 dpf, vMCs progressively cover SeAs but not SeVs (arrows). The graph shows the percentage of SeAs covered by vMCs in the trunk. By 5 dpf, supraintestinal arteries (SIAs) are also covered by vMCs (yellow arrows). Data are represented as mean ± SD. Scale bar, 50 μm. (D) Confocal transverse sections of the trunk region of Tg(*kdrl:egfp*)^s843^ embryos stained for Tagln (red). vMCs are located only around SeAs but not SeVs. Blue, nuclei. Scale bar, 25 μm. (E) Quantification of vMC coverage in arterial and venous zebrafish vessels. Double Tg *Tg(flt1:Mmu.Fos-EGFP)*^*wz2*^;*Tg(−5.2lyve1b:DsRed)*^*nz101*^ zebrafish embryos expressing EGFP in arteries and dsRed2 in veins (Nicenboim et al., 2015) were stained with the vMC marker Tagln (gray) of 4 dpf. A total number of five z-stacks for 10 embryos were analyzed. Histograms show the percentage of vessels covered by vMCs compared to the total number counted for each category. No vMCs were found around veins in all experimental conditions. Data are represented as mean ± SD. (F) vMCs are located only around arterial endothelial cells (arrows). Partial z-projection of the trunk region (somite 8–14) of *Tg(acta2:mCherry)*^*uto5*^; *Tg(tp1:egfp)*^*um14*^. Merged and single confocal channels are shown. Scale bar, 100 μm. (G) Confocal transverse sections of the trunk region of *Tg(tp1:egfp)*^*um14*^ stained for Tagln (red). vMCs are positioned only around *tp1*-positive SeA vessels (arrows). Blue, nuclei. Scale bar, 25 μm. See also Figures S1 and S2.

We previously showed that zebrafish vMCs share many of the morphological, molecular, and functional characteristics of mammalian vMCs, making the zebrafish a useful model to study the mechanisms of mural cell recruitment and differentiation (Santoro et al., 2009). To better study vascular myogenesis in zebrafish, we generated zebrafish Tg lines marking vMCs at very early stages. These Tg lines express the fluorescent markers *mCherry* and a membrane-localized *egfp* under the control of two early mural cells markers: a minimal promoter region for *acta2* (*αSMA* [smooth muscle actin α]) and *tagln* (*SM22α−b*) (Figures S1A and S1B). Bona fide expression of these Tg lines for vMCs is shown by the expression of the fluorescent markers in cells surrounding vessels located in the eye and ventral aorta as well as trunk vasculature, as previously shown (Figures S1C and S1D) (Fortuna et al., 2015, Georgijevic et al., 2007, Santoro et al., 2009, Wang et al., 2014, Whitesell et al., 2014). To shed light on the process of vMC recruitment and coverage of blood vessels, we imaged the trunk and brain vasculature, starting from 60 hpf (hour post fertilization). These are also previously established conditions for detecting vMCs during zebrafish development using different mural cell/pericyte-specific markers (Georgijevic et al., 2007, Santoro et al., 2009). By crossing these Tg lines with the endothelial-specific lines *Tg(kdrl: egfp)*^*s843*^ or *Tg(kdrl:CAAX-mCherry)*^*uto2*^, we analyzed the distribution of vMCs in the trunk vessels as well as the hindbrain region of a developing zebrafish embryo. Confocal analyses of live transgenic embryos have shown that vMCs are present around the dorsal aorta (DA), but not around the cardinal vein (CV) (Figure 1A). These vMCs are mainly attached to the ventral side of the DA (Figure 1B), as previously described (Santoro et al., 2009). vMCs have also been later found in contact with intersomitic vessels (Ses) of the trunk (Figure 1C). However, we noticed that only arterial-fated Ses (SeAs) are covered by vMCs, whereas vein-fated Ses (SeVs) are specifically excluded in this process. A confocal analysis of embryonic vasculature cross sections confirmed these results by showing that only the arterial vessels arising from the DA (SeAs) are surrounded by vMCs (Figure 1D). In addition, we also analyzed hindbrain vasculature. In agreement with previous observations in the trunk vasculature, we observed that vMCs mainly cover arterial vessels in the brain (Figures S2A–2D). To quantify vMC coverage of arterial- and venous-fated vessels, we performed Tagln staining in *Tg(flt1:Mmu.Fos-EGFP)*^*wz2*^;*Tg(−5.2lyve1b:DsRed)*^*nz101*^ to precisely locate arterial and venous vessels (Figure 1E) (Nicenboim et al., 2015). These data show that DA and SeAs are covered by Tagln-positive cells, whereas vein-fated vessels, such as the posterior cardinal vein (PCV) and SeVs, are not. The same occurred in the head region (Figure S2E). To further support the observation that vMCs mainly covered arterial-fated vessels, we crossed our vMC Tg lines with a reporter line that patterns the arterial vascular system, such as *Tg(tp1:egfp)*^*um14*^ (Quillien et al., 2014). Here, we specifically detected vMC coverage only for arterial-fated *tp1:egfp*-positive vessels and throughout the vascular bed (Figure 1F). Similar results have been obtained by staining vMCs with Tagln antibody in the *Tg(tp1: egfp)*^*um14*^ background (Figure 1G). Taken together, these data clearly show that vMCs are recruited and attach exclusively to arterial-fated vessels at early stages of zebrafish vascular development.

### Blood Flow and Hemodynamic Forces Regulate vMC Recruitment

**Figure 2:**
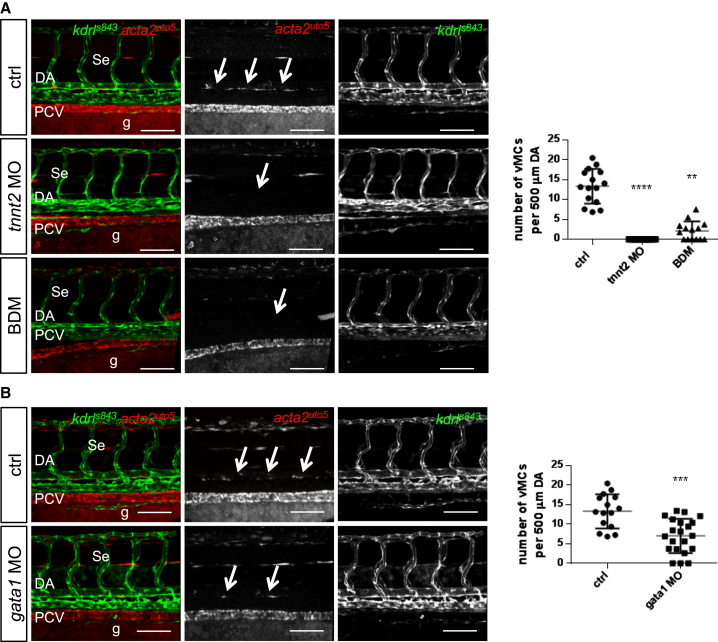
Blood Flow and Hemodynamic Forces Regulate vMC Recruitment (A) Genetic and pharmacological impairment of blood flow prevent vMC recruitment. Confocal images of partial z-projections of the trunk region (somite 8–14) of *Tg(kdrl:egfp)*^*s843*^*;Tg(acta2:mCherry)*^*uto5*^ embryos at 3 dpf (merged and single channels) after injection of *tnnt2* morpholinos or 2,3-BDM treatment. Compared to controls, vMCs (arrows) are not recruited around the DA of embryos with impaired blood flow. Scatter plots show the quantification of vMC number per 500 μm of the DA at 80 hpf. n = 15, 26, and 15 embryos, respectively. Data are represented as mean ± SD. Stars represent the results of one-way ANOVA-Dunnett’s post hoc test (^∗^p < 0.05, ^∗∗^p < 0.01, ^∗∗∗^p < 0.001, ^∗∗∗∗^p < 0.0001). g, gut. (B) Abrogation of erythrocytes and reduced shear stress impair vMC coverage. Confocal images of partial z-projections of the trunk region (somite 8–14) of *Tg(kdrl:egfp)*^*s843*^*;Tg(acta2:mCherry)*^*uto5*^ embryos at 3 dpf (merged and single channels) after *gata1* knockdown by morpholino injection. Compared to controls, *gata1* morphants showed reduced vMCs around the DA (arrows). Scatter plots show the quantification of vMC number per 500 μm of the DA at 80 hpf. n = 15 and 23 embryos, respectively. Data are represented as mean ± SD. Stars represent the results of unpaired t tests of mean difference = 0 (^∗^p < 0.05, ^∗∗^p < 0.01, ^∗∗∗^p < 0.001). See also Figure S3.

The function of shear stress and hemodynamic forces in vascular development has been investigated for many years as a critical mechanism in shaping the developing vascular system (Boselli et al., 2015). In particular, it has been shown that shear stress induced by blood flow is a critical regulator of arteriogenesis and vascular homeostasis supporting Notch expression in early vascular development and Notch activation in endothelial progenitors (Jahnsen et al., 2015, Obi et al., 2009). However, whether shear stress is important for vascular myogenesis in vertebrates remains unknown. We therefore investigated whether shear stress is required for vMC recruitment and differentiation in zebrafish embryos. To do so, we ablated blood flow in developing embryos by treating embryos with 2,3-butanedione monoxime (BDM) or knocking down *tnnt2* (*troponin T type 2, cardiac*), a gene required for cardiac function (Nicoli et al., 2010). Both pharmacological and genetic blockade of blood flow selectively showed impairment of vMC coverage of arterial vessels (Figures 2A and S3). To further isolate the effect of shear stress on mural cell recruitment versus effects on the vasculature as a whole, we also assessed vMC recruitment in a model of reduced shear stress by depletion of blood erythrocytes using knock down of *gata1* (*globin transcription factor 1*), a gene required for erythroblast development that does not affect vessel homeostasis (Boselli et al., 2015, De Luca et al., 2014). Here, we found that reduced hemodynamic forces reduced vMC coverage around arterial-fated vessels compared to controls (Figures 2B and S3). These data indicate that blood flow is required for vMC coverage during zebrafish vascular development.

### Arterial Notch Signaling Is Required for vMC Recruitment

**Figure 3:**
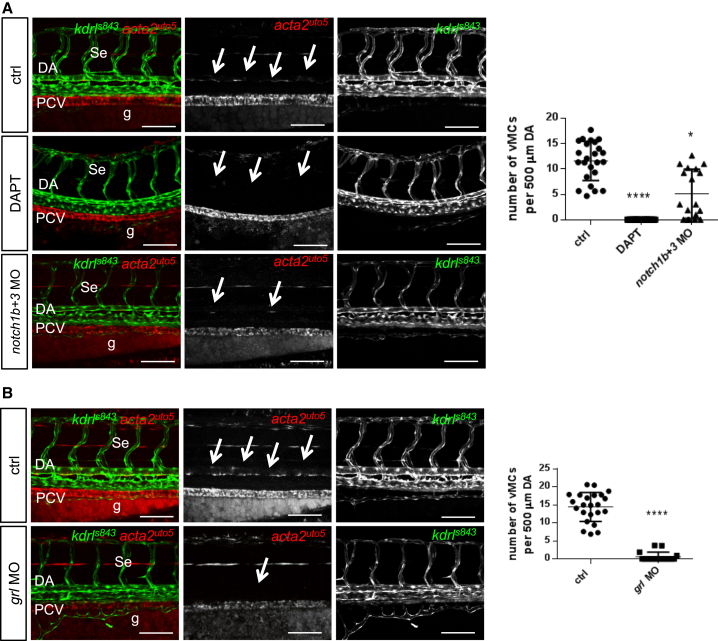
Notch Activation Is Required for Arterial vMC Recruitment (A) Pharmacological and genetic inhibition of Notch signaling reduces vMC recruitment around the DA. Confocal images of partial z-projections of the trunk region (somite 8–14) of *Tg(kdrl:egfp)*^*s843*^*Tg(acta2:mCherry)*^*uto5*^ embryos at 80 hpf (merged and single channels) treated with DAPT or co-injected with *notch1b* and *notch3* morpholinos. Scatter plots show the number of vMCs counted along 500 μm of the DA at 80 hpf. n = 27, 29, and 19 embryos. Data are represented as mean ± SD. Stars represent the results of one-way ANOVA-Dunnett’s post hoc test (^∗^p < 0.05, ^∗∗^p < 0.01, ^∗∗∗^p < 0.001, ^∗∗∗∗^p < 0.0001). g, gut. (B) *Hey2/grl* is required for vMC recruitment. Confocal images of partial z-projections of the trunk region (somite 8–14) of *Tg(kdrl:egfp)*^*s843*^*;Tg(acta2:mCherry)*^*uto5*^ embryos at 80 hpf (merged and single channels) after *grl* knockdown by morpholinos injection. Compared to controls, *grl* morphants lack vMC coverage around the DA (arrows). Scatter plots show the quantification of vMC number per 500 μm of the DA at 3 dpf. n = 23 and 17 embryos. Data are represented as mean ± SD. Stars represent the results of unpaired t tests of mean difference = 0 (^∗^p < 0.05, ^∗∗^p < 0.01, ^∗∗∗^p < 0.001, ^∗∗∗∗^p < 0.0001). See also Figure S4.

Extensive studies have shown that specific signaling programs are exclusively activated in arteries compared to veins (Herbert and Stainier, 2011, Lawson et al., 2001). In particular, the Notch signaling pathway plays a central role in the expression of artery-specific genes (i.e., *ephrin-B2*) and repression of venous markers (i.e, *ephB4*) within developing arteries (Lawson et al., 2001, Siekmann and Lawson, 2007a). We therefore decided to investigate whether Notch is required for vMC recruitment in arterial-fated vessels. To do so, we treated double *Tg(kdrl:egfp)*^*s843*^*; Tg(acta2:mCherry)*^*uto5*^ embryos with the Notch inhibitor (N-[N-(3,5-difluorophenacetyl)- L-alanyl]-S-phenylglycine t-butyl ester) (DAPT). To avoid pleiotropic effects on the vasculature, we inhibited Notch signaling only after arterial-vein differentiation had taken place. The DAPT treatment was able to completely abrogate vMC recruitment around trunk vasculature (Figures 3A and S4). In addition, concomitant gene inactivation of *notch1b* and *notch3* caused a reduction of vMC coverage in the trunk vasculature (Figures 3A and S4A). In contrast, single *notch1b* or *notch3* gene inactivation did not exert any effect on vMC coverage (Figure S4B), most likely due to the compensation effect of multiple Notch receptors for artery differentiation (Quillien et al., 2014). Lastly, to test the essential role of arterial differentiation in vascular myogenesis, we analyzed the function of *grl*/*hey2* in vMC coverage in zebrafish. *grl*/*hey2* (*hairy and enhancer of split-related 2*) is a downstream effector of Notch signaling in vertebrates, with a role in arterial differentiation in zebrafish (Swift and Weinstein, 2009, Zhong et al., 2001). We found that the blockade of *grl* expression in zebrafish embryos specifically impairs vMC recruitment (Figures 3B and S4C).

Our results indicate that Notch signaling and endothelial arterialization are required to promote vascular myogenesis in developing zebrafish embryos.

### Arterial Notch Signaling Acts Downstream of Flow to Recruit vMCs

**Figure 4:**
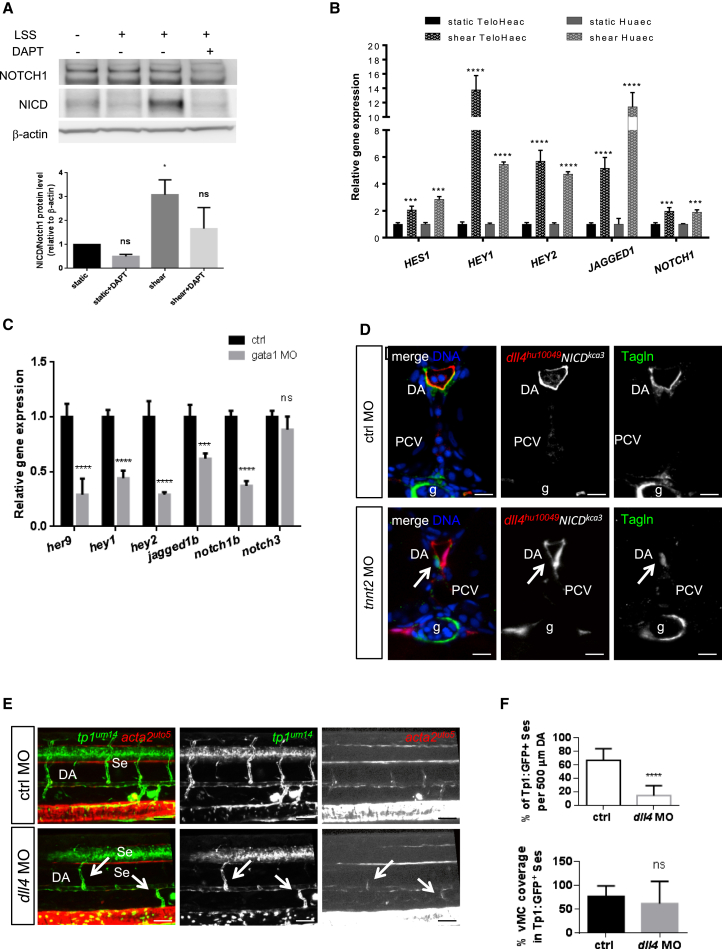
Notch Signaling Acts Downstream of Blood Flow to Recruit vMCs (A) Shear-stress-mediated Notch activation in arterial-fated endothelial cells. Western blot analyses show increased levels of cleaved intracellular Notch (NICD) after 4 hr of LSS, whereas total Notch1 is unchanged. β-actin has been used as loading control. Histograms show the quantification of the amount of cleaved Notch1 (NICD)/over total Notch1 upon shear stress in endothelial cells. (B) Shear stress induces Notch signaling in ECs. Effects of shear stress on the expression of different Notch target genes in human endothelial cell lines after 24 hr of LSS. ^∗∗∗^p < 0.001, ^∗∗∗∗^p < 0.0001. Data are represented as mean ± SD. Stars represent the results of two-way ANOVA. (C) Loss of Notch signaling by blockade of blood flow in living zebrafish vessels. qPCR analyses of different Notch target genes are downregulated in ECs sorted from *Tg(kdrl:egfp)*^*s843*^ injected with control or *gata1* MO at 48 hpf. ^∗∗∗^p < 0.001; ^∗∗∗∗^p < 0.0001. Data are represented as mean ± SD. Stars represent the results of two-way ANOVA. (D) Arterial Notch activation restores vMC recruitment in the absence of blood flow in zebrafish vessels. Confocal images of Tagln staining (green) on section of *TgBAC(dll4:GAL4FF;UAS:RFP)*^*hu10049*^*Tg(UAS:NICD)*^*kca3*^ injected with *tnnt2* or control morpholino. Expression of NICD in arterial ECs (*dll4*:RFP) rescue vMC recruitment (arrow) in the absence of blood flow (*gata1* morphants). All the embryos analyzed (n = 15 in both conditions) show the indicated phenotype. Blue, nuclei. Scale bar, 25 μm. (E) Blood flow can promote Notch signaling and vMC coverage in arterial vessels in the absence of *dll4*. Injection of *dll4* morpholino induces a reduction of *EGFP*-positive Se vessels in *Tg(tp1:egfp)*^*um14*^ embryos. The few Se vessels that are positive for Notch activity and vMC coverage are the ones with circulation (arrow). A total number of 23 controls and 30 morphants were analyzed. Scale bar, 50 μm. (F) Notch signaling is impaired after *dll4* inactivation (KD). Histograms show the percentage of *tp1:GFP*^+^ Se vessels in 500 μm of DA, which are reduced in *dll4* MO (n = 22/155 Se) compared to controls (n = 85/128 Se). The coverage of *tp1+* vessels by vMCs is not altered in *dll4* MO (n = 9/22) compared to controls (55/85), showing that flow can still recruit vMCs. A total number of 23 controls and 30 morphants were analyzed. Data are represented as mean ± SD. Stars represent the results of unpaired t tests of mean difference = 0 (^∗^p < 0.05, ^∗∗^p < 0.01, ^∗∗∗^p < 0.001). Arrows indicate vessels with blood flow in *dll4* morphants. See also Figures S5 and S6.

Next, we hypothesized that hemodynamic forces might be important for vMC recruitment by modulating Notch signaling in endothelial cells. Under physiological conditions, shear stress in the human aorta ranges from 10 to 20 dynes/cm^2^, whereas a shear stress of 1–6 dynes/cm^2^ acts on the walls of the vein (Ando and Yamamoto, 2013). We exposed human endothelial cells to laminar shear stress (LSS) resembling arterial conditions (15 dynes/cm^2^) and tested for Notch activation using western blot with an NICD (Notch1 intracellular domain) antibody. We observed that shear stress can promote NICD activation after 4 hr of LSS (Figure 4A). Furthermore, we evaluated the expression levels of Notch target genes in arterial ECs subjected to LSS. qRT-PCR analyses on two types of human arterial ECs showed that all the Notch target genes analyzed were significantly induced by LSS (Figure 4B). These data strongly support a mechanism of Notch signaling activation driven by shear stress in arterial ECs. To validate these findings in vivo, we analyzed the expression of Notch signaling pathway genes in fluorescence-activated cell sorting (FACS)-sorted zebrafish ECs exposed to reduced hemodynamic forces as in *gata1* morphants. qRT-PCR analyses revealed that all Notch target genes were downregulated in the absence of normal blood flow (Figure 4C).

To prove that shear-stress-induced endothelial Notch signaling is responsible for vMC coverage in vivo, we attempted to rescue vMC recruitment in flow-impaired embryos (e.g., *tnnt2* and *gata1* morphants) by expressing NICD in ECs using the arterial *dll4:GAL4FF* Tg line (Hermkens et al., 2015) and the *UAS:NICD* Tg line (Scheer and Campos-Ortega, 1999). We found that arterial *dll4*-regulated expression of NICD can specifically rescue vMC recruitment in the absence of hemodynamic forces (Figures 4D and S5). In addition, we analyzed the effects of *dll4* downregulation in zebrafish embryos. We observed that in *dll4* morphants, whereas arterial and venous differentiation is not affected (Figure S6) (Lawson et al., 2001), there are severe circulation defects in segmental vessels, with few SeAs able to carry blood flow (Movie S1). Analysis of the *dll4* knock-down (KD) in the *Tg(tp1:EGFP)^um14^* line shows that only those SeAs with circulating blood flow are *tp1* positive and also covered by vMCs (Figures 4E and 4F). These data indicate that in the absence of the Notch ligand *dll4*, blood flow per se can support Notch activation and leads to vMC recruitment.

These data strongly indicate the existence of an exclusive flow-dependent mechanism that leads to Notch activation in arteries and that is required for vMC recruitment in developing zebrafish vasculature.

### Primary Cilia Are Required for vMC Recruitment by Regulating Shear-Stress-Dependent Notch Activation

**Figure 5:**
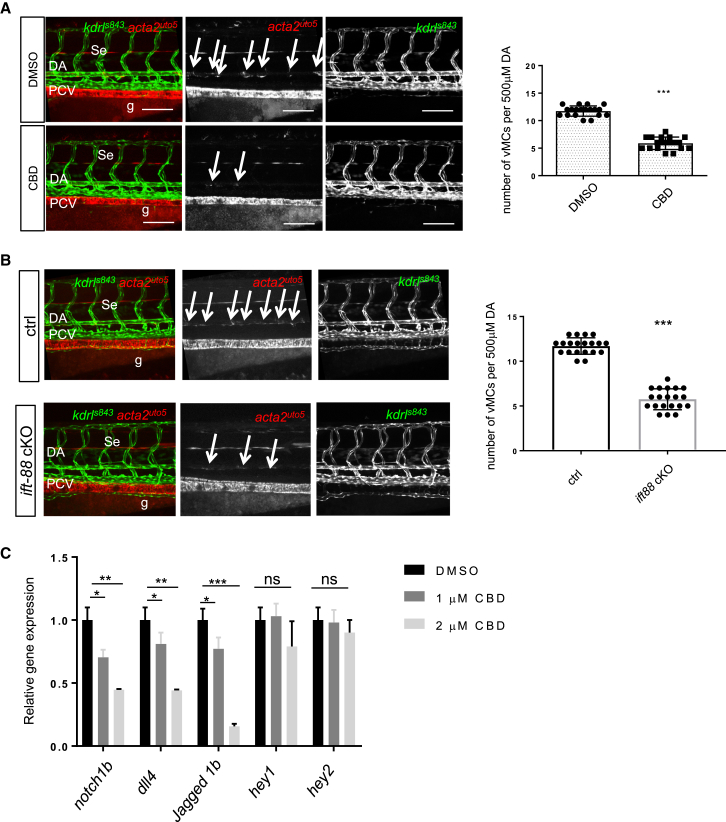
Cilia Are Required to Drive vMC Recruitment (A) Pharmacological impairment of primary cilia formation negatively regulates vMC recruitment. Confocal images of partial z-projections of the trunk region (somite 8–14) of *Tg(kdrl:egfp)*^*s843*^*;Tg(acta2:mCherry)*^*uto5*^ embryos at 80 hpf (merged and single channels) after disrupting cilia formation by CBD treatment. Compared to controls, CBD-treated embryos show reduced vMC coverage around the DA (arrows). Scatter plots show the quantification of vMC number per 500 μm of the DA at 80 hpf. n = 39 and 53 embryos. Data are represented as mean ± SD. Stars represent the results of unpaired t tests of mean difference = 0 (^∗^p < 0.05, ^∗∗^p < 0.01, ^∗∗∗^p < 0.001, ^∗∗∗∗^p < 0.0001). g, gut. (B) Endothelial-specific genetic inhibition of *ift88* gene expression impairs vMC coverage. Confocal images of partial z-projections of the trunk region (somite 8–14) of *Tg(kdrl:egfp)*^*s843*^*Tg(acta2:mCherry)*^*uto5*^ embryos at 80 hpf (merged and single channels) injected with the integratable CRISPR vector for endothelial-specific *ift88* gene targeting. Cmlc2:GFP-negative embryos were used a negative control (ctrl). Scatter plots show the quantification of vMC number per 500 μm of the DA at 80 hpf. n = 20 and 20 embryos. Data are represented as mean ± SD. Stars represent the results of unpaired t tests of mean difference = 0 (^∗^p < 0.05, ^∗∗^p < 0.01, ^∗∗∗^p < 0.001, ^∗∗∗∗^p < 0.0001). (C) Inhibition of cilia function by CBD treatment impairs Notch signaling in living zebrafish blood vessels. qPCR analysis of Notch target genes in DMSO and 1 and 2 μM CBD-treated embryos. qPCR analysis of Notch target gene expression in zebrafish treated with different concentrations of CBD at 48 hpf. A zebrafish trunk was used for RNA extraction and qPCR analyses. *Notch1b*, *dll4*, and *Jagged1b* are the most affected Notch targets by the lack of cilia at this stage of development (48 hpf). Data are represented as mean ± SD. Stars represent the results of two-way ANOVA. ^∗^p < 0.05, ^∗∗^p < 0.01, ^∗∗∗^p < 0.001. See also Figures S7–S10.

It has been shown that zebrafish endothelial cells sense mechanical forces from blood flow through primary cilia (Goetz et al., 2014, Kallakuri et al., 2015). To test whether primary cilia play a key role in flow-dependent mechano-activation of Notch signaling and vMC recruitment, we used a pharmacological approach, the AAA+ ATPase motor cytoplasmic dynein inhibitor ciliobrevin D (CBD). CBD is known to reduce the microtubule cycling necessary to construct and maintain primary cilia and is commonly used to impair cilia function (Firestone et al., 2012, Samsa et al., 2015). We injected the pericardium of *Tg(acta2:cherry)*^*uto5*^ embryos with CBD or DMSO at 54 hpf and assessed vMC recruitment at 80 hpf (Figure 5A). Although injection of DMSO has no effect, embryos injected with CBD show a significant reduction in the number of vMCs surrounding the DA. We also found that the effect of the cilia inhibitor CBD on vMC recruitment is dose dependent (Figure S7). By treating embryos with CBD before (56 hpf) or after (72 hpf) the onset of the appearance of vMC, we found that CBD treatment blocks vMC recruitment only if the treatment occurs before the onset of vMC recruitment, confirming that primary cilia are required before the appearance of vMCs and are not related to vMC homeostasis (Figure S8). To better characterize the cell autonomous role of endothelial primary cilia, we exploited genetic experiments by CRISPR technology. Taking advantage of a CRISPR/Cas9 vector system for tissue gene disruption in zebrafish (Ablain et al., 2015), we inactivated *ift88* selectively in zebrafish endothelial cells (Figures 5B and S9). Ift88 is a critical protein in ciliogenesis, and its deletion is known to cause ciliopathies in zebrafish (Kramer-Zucker et al., 2005, Tsujikawa and Malicki, 2004, Goetz et al., 2014, Samsa et al., 2015). Zebrafish embryos carrying an *ift88* deletion in developing vascular tissues show severe impairment in vMC recruitment. This occurs in the context of normal vascular development and overall normal blood vessel morphology. These data indicate that primary cilia control vascular myogenesis in developing zebrafish embryos.

Primary cilia are known to activate or contribute to the activation of specific signaling events inside cells (Goetz and Anderson, 2010). We next asked whether primary cilia play a key role in vMC recruitment by affecting Notch signaling in living blood vessels. We analyzed expression of Notch target genes in CBD-treated embryos. qRT-PCR analyses reveal that Notch target genes are downregulated in the absence of normal primary cilia in developing embryos (Figure 5C). We also tested and discarded the possibility that cilia impair Notch-mediated artery-vein differentiation by analyzing *ephrinB2* and *flt4* expression in CBD embryos (Figure S10).

Overall, these findings indicate that endothelial primary cilia are indispensable for driving vascular maturation, leading to vMC recruitment and differentiation in zebrafish embryos. These data also suggest that the arterial Notch signaling required for vMC recruitment and differentiation may be regulated by primary cilia. We thus speculate that primary cilia may represent the mechano-transducers responsible for flow-dependent Notch signaling activation in ECs and vascular tissue.

### Expression of the FoxC Family Member *foxc1b* Is Flow and Cilia Dependent and Required for Vascular Myogenesis

**Figure 6:**
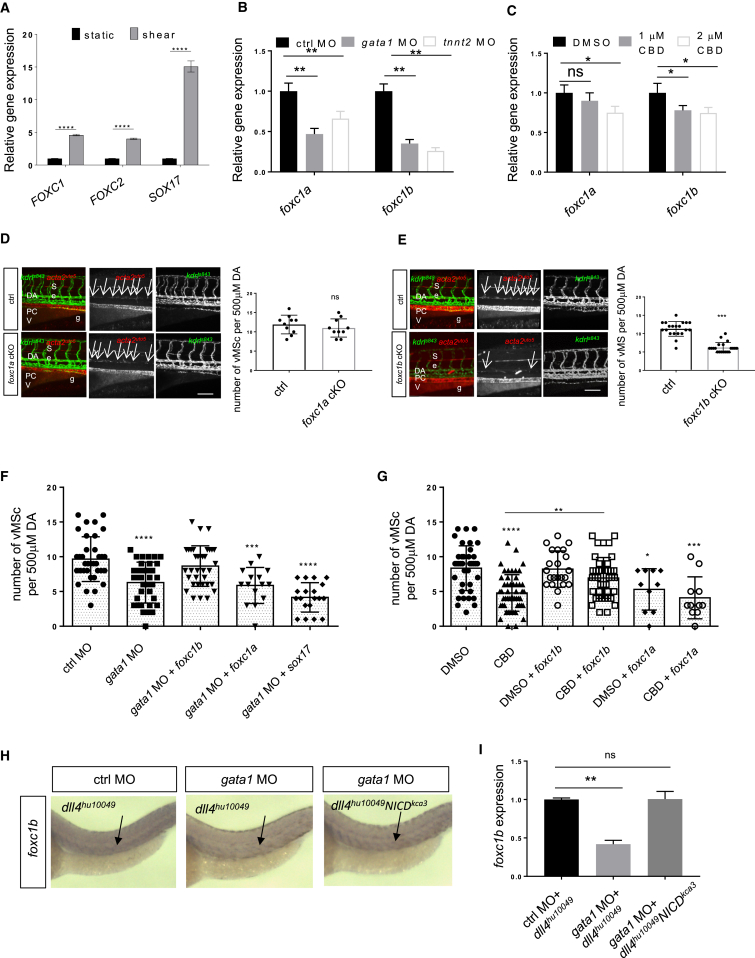
*foxc1b* Required Downstream Cilia and Notch Signaling to Recruit vMCs around Developing Vasculature (A) Shear stress modulates expression of *FOXC1* and *FOXC2*. Arterial endothelial cells exposed to 24 hr of LSS show increased expression of *FOXC1*, *FOXC2*, and *SOX17* genes. Data are represented as mean ± SD. Stars represent the results of one-way ANOVA. ^∗∗∗∗^p < 0.00011. (B) Blockade of shear stress in zebrafish inhibits *foxc1a* and *foxc1b* gene expression. qPCR analysis of *foxc1a* and *foxc1b* gene expression in zebrafish injected with control MO, *gata1* MO, and *tnnt2* MO. *Foxc1b* is more affected by the lack of flow compared to *foxc1a*. Data are represented as mean ± SD. Stars represent the results of two-way ANOVA. ^∗∗^p < 0.01. (C) Primary cilia impairment inhibits zebrafish *foxc1a* and *foxc1b* gene expression. qPCR analysis of *foxc1a* and *foxc1b* gene expression in zebrafish control and 1 or 2 μM CBD-treated embryos at 48 hpf. The trunk of treated embryos was used for RNA extraction and qPCR analyses. *Foxc1b* is more affected by the lack of cilia compared to *foxc1a*. Data are represented as mean ± SD. Stars represent the results of two-way ANOVA. ^∗^p < 0.1. (D) Endothelial-cell-specific *foxc1a* inhibition reduces vMC recruitment around the DA. Confocal images of partial z-projections of the trunk region (somite 8–14) of *Tg(kdrl:egfp)*^*s843*^*;Tg(acta2:mCherry)*^*uto5*^ embryos at 80 hpf (merged and single channels) injected with endothelial-cell-specific *foxc1a* cKO plasmid together with Tol2 mRNA. Cmlc2-negative embryos were used a control (ctrl). Asterisks indicate acta2-positive somites in cmlc2:GFP-positive embryos. n = 10 and 10 embryos. Right: scatter plots show the number of vMCs counted along 500 μm of the DA. ns indicates no significant difference in a one-way ANOVA test. Data are represented as mean ± SD. (E) Endothelial-cell-specific *foxc1b* inhibition reduces vMC recruitment around the DA. Confocal images of partial z-projections of the trunk region (somite 8–14) of *Tg(kdrl:egfp)*^*s843*^*Tg(acta2:mCherry)*^*uto5*^ embryos at 80 hpf (merged and single channels) injected with endothelial-cell-specific *foxc1b* cKO plasmid together with Tol2 mRNA. Cmlc2:GFP-negative embryos were used as control (ctrl); n = 20 and 20 embryos. Right: scatter plots show the number of vMCs counted along 500 μm of the DA. Data are represented as mean ± SD. Stars represent the results of two-way ANOVA. ^∗∗∗^p < 0.001. (F) *Foxc1b*, but not *foxc1a* or *sox17*, expression restores vMC recruitment in absence erythrocytes and reduced shear stress in zebrafish vessels. Scatter plots show the quantification of vMC number per 500 μm of the DA obtained from confocal images of partial z-projections of the trunk region (somite 8–14) of *Tg(kdrl:egfp)*^*s843*^*Tg(acta2:mCherry)*^*uto5*^ embryos at 80 hpf after *gata1* morpholino injection alone or with mRNA of *foxc1b*, *foxc1a*, or *sox17*, respectively. Compared to *gata1* morphants, coinjection of *foxc1b* showed restored vMC recruitment around the DA. n = 34, 35, 34, 15, and 19 embryos, respectively. Data are represented as mean ± SD. Stars represent the results of one-way ANOVA-Dunnett’s post hoc test. ^∗^p < 0.05, ^∗∗^p < 0.01, ^∗∗∗^p < 0.001, ^∗∗∗∗^p < 0.0001. (G) vMC recruitment is rescued by expression of *foxc1b* in a zebrafish embryo chemically inhibited for cilia formation. Scatter plots show the quantification of vMC number per 500 μm of the DA obtained from confocal images of partial z-projections of the trunk region (somite 8–14) of *Tg(kdrl:egfp)*^*s843*^*Tg(acta2:mCherry)*^*uto5*^ embryos at 80 hpf. After injection of the mRNA of *foxc1b*, *foxc1a*, or *sox17* at one single-cell stage, embryos were again injected in the pericardium with CBD or DMSO at 54 hpf. Only embryos carrying exogenous *foxc1b* expression showed restored vMC recruitment around the DA after cilia impairment. n = 40, 52, 21, 41, 9, and 10 embryos, respectively. Data are represented as mean ± SD. Stars represent the results of one-way ANOVA-Dunnett’s post hoc test (^∗^p < 0.05, ^∗∗^p < 0.01, ^∗∗∗^p < 0.001, ^∗∗∗∗^p < 0.0001). (H) *foxc1b* expression is downstream of Notch signaling. Representative images of whole-mount in situ hybridization of *foxc1b* gene in zebrafish *Tg(dll4:Gal4;UAS:mCherry;UAS:NICD)* embryos at 48 hpf injected with control or *gata1* morpholino. Arrows show specific *foxc1b* expression. Ten embryos for each condition were analyzed, and all of them showed the specific phenotype. (I) Arterial Notch activation (NICD) rescues flow-dependent impairment of *foxc1b* expression. qPCR analysis of *foxc1b* gene expression in zebrafish *Tg(dll4:Gal4;UAS:mCherry;UAS:NICD)* embryos at 48 hpf injected with control or *gata1* morpholino. Reduced *foxc1b* expression in *gata1* morphants is fully rescued by arterial NICD expression. Data are represented as mean ± SD. Stars represent the results of one-way ANOVA-Dunnett’s post hoc test (^∗∗^p < 0.01). See also Figures S11–S13.

Previous work in mammals has identified the FoxC family of Forkhead proteins as essential for vascular homeostasis and differentiation. In particular, FoxC1 and FoxC2 have been found to play critical functions in ECs during arterial (and lymphatic) differentiation by acting as important downstream effectors of Notch signaling (De Val and Black, 2009, Seo et al., 2006). These data suggest that the FoxC family functions relatively late during vascular development, making them good candidates for being involved in vascular maturation and vMC recruitment. To test this hypothesis, we first analyze whether *FOXC1* and *FOXC2* are activated by LSS in arterial ECs (Figure 6A). We discovered that both *FOXC1* and *FOXC2* are induced by shear stress. As a control, we also analyzed another gene critical for arterialization, *SOX17*, which is also induced by LSS. We then analyzed whether shear-stress-mediated FOXC1 and FOXC2 expression might be dependent on blood flow and cilia in vivo. We analyzed expression of *foxc1a* and *foxc1b* (zebrafish ortholog of mammalian *FOXC1* and *FOXC2*, respectively) in blood-flow- and cilia-impaired embryos (*gata1* morphants and CBD-treated embryos, respectively) (Figures 6B and 6C). Consistent with previous studies (Jang et al., 2015, Kim et al., 2014, Skarie and Link, 2009), zebrafish *foxc1a* is expressed in the arteries and veins, whereas *foxc1b* is expressed only in arteries and Ses during development (Figure S11). qRT-PCR analyses on zebrafish embryos in the absence of flow or primary cilia revealed that *foxc1a* and *foxc1b* genes are both downregulated in these conditions, further supporting the hypothesis that endothelial primary cilia might modulate Notch signaling and support *foxc1a/b* expression for vMC recruitment in living blood vessels.

We investigated the role of zebrafish FoxC proteins in zebrafish vascular myogenesis by inactivating *foxc1a* and *foxc1b* expression in developing endothelial cells using the CRISPR/Cas9 system (Figure S10). We analyzed vMC recruitment and differentiation after endothelial-specific knock-down of *foxc1a* and *foxc1b*, respectively (Figures 6D and 6E). Both *foxc1a* and *foxc1b* EC-specific mutant embryos show normal vascular development, as confirmed by previous morpholino experiments (Jang et al., 2015). However, only *foxc1b* mutants show a statistically significant impairment of vMC coverage. These data indicate that Foxc1b is required for vascular mural cell recruitment and maturation in zebrafish, a role that may be conserved.

To prove that *foxc1b*, but not *foxc1a*, is critical for vascular myogenesis and that its function is downstream and dependent on blood flow, we tested whether expression of *foxc1a* or *foxc1b* mRNA rescues *gata1* morphants (Figure 6F). As a control, *sox17* mRNA was used because we found that it can be induced by shear stress in vitro and is expressed in the zebrafish vasculature (data not shown; Hermkens et al., 2015). Interestingly, expression of *foxc1b*, but not *foxc1a* or *sox17*, can rescue the lack of vMC recruitment in *gata1* morphants. Next, we tested whether *foxc1b* can rescue the absence of vMCs induced by cilia impairment (Figure 6G). Again, expression of *foxc1b*, but not of *foxc1a*, rescues the lack of vMC recruitment in CBD-treated embryos. Overall, we can conclude that *foxc1b*, but not *foxc1a*, leads to vMC recruitment and differentiation by acting downstream of flow and cilia.

Because FoxC proteins have been associated with arterial differentiation in mice, we tested whether *foxc1b* downregulation alters A-V differentiation and, therefore, vMC arterial coverage. We analyzed arterial and venous markers in flow-impaired *gata1* morphants as well as *foxc1b* mutant embryos and morphants (Figure S12). We did not score any alteration in *ephrinB2* and *flt4* expression, suggesting that flow or cilia and *foxc1b* function after A-V differentiation in zebrafish embryos.

Previous data indicated that FoxC proteins act downstream of Notch signaling (De Val and Black, 2009). To test whether *foxc1b* is induced by Notch in a flow-dependent manner, we analyzed *foxc1b* expression in *gata1* morphants expressing NICD in arterial ECs using the *TgBAC(dll4:GAL4FF;UAS:RFP)*^*hu10049*^ crossed with *Tg(UAS:NICD)*^*kca3*^ (Figures 6H and 6I). We found that arterial *dll4*-driving expression of NICD can specifically rescue *foxc1b* expression in the absence of hemodynamic forces. *foxc1a* does not behave in the same manner (Figure S13). These genetic data fit and corroborate the previous functional data supporting a critical role for *foxc1b* during vascular myogenesis in zebrafish. Altogether, these data suggest that *foxc1b* (but not *foxc1a*) is a critical gene responsible for vMC recruitment downstream of cilia-dependent Notch activation.

## Discussion

**Figure 7:**
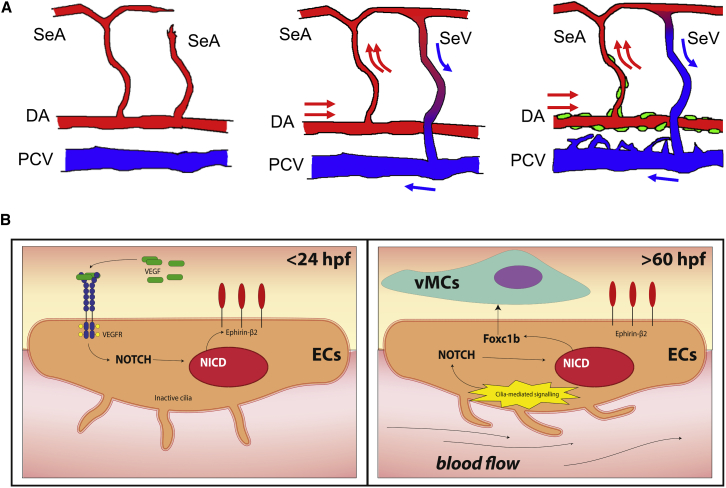
Schematic Representation of Vascular Myogenesis and Their Molecular Mechanisms (A) Schematic model of vascular myogenesis during zebrafish vascular development. In zebrafish, angiogenic cell behavior is initially restricted to arterial ECs, which form intersegmental vessels (ISVs) in the trunk under the control of Vegf and Notch signaling (Lawson et al., 2002) (left panel). Afterward, venous angiogenesis takes place under the control of *vegfc*, *flt4*, and *ccbe1* (van Impel et al., 2014). All emerging sprouts from the PCV migrate dorsally, with some of them connecting to Se and becoming SeV. The others remain SeAs (middle panel). Once this remodeling is completed, only arterial blood vessels start to recruit vMCs (right panel). We can speculate that a different type of flow between arteries and veins at this stage of development (e.g., pulsatile versus steady flow; [Anton et al., 2013]) might be responsible for a discrete Notch signaling requirement, leading to selective vMC recruitment of only arterial-fated vessels. Arrows indicate blood flow. (B) Molecular mechanism activated by blood flow and cilia during vascular myogenesis. Before the onset of circulation, VEGF signaling activates the Notch pathway to promote arterial differentiation and EphB2 expression (Lawson and Weinstein, 2002). After arterialization, mechanical stress induced by blood flow activates primary cilia-mediated intracellular signaling. This in turn activates Notch signaling, which promotes expression of *foxc1b*. *foxc1b* expression in the endothelium is sufficient to drive vMC recruitment by activating mesodermal cells present in closed to arterial-fated vessels (Santoro et al., 2009).

The formation of a functional cardiovascular system is driven by a complex interaction among hemodynamic forces, genetic programs, and cell-cell interactions. The role of blood flow and shear stress in vascular maturation and mural cells/smooth muscle recruitment has been little investigated in vertebrates. Here, we use the zebrafish model system to investigate how these factors are functionally connected and might control arterial maturation and vascular myogenesis. Through genetic and pharmacological approaches, we find that vascular myogenesis occurs in zebrafish embryos after arterial-vein differentiation. Arterially fated vessels can provide molecular clues to mesodermal cells to migrate and differentiate into vascular mural cells and adhere to the developing blood vessels (Santoro et al., 2009). Arterial differentiation, although necessary, is not sufficient to control vascular myogenesis because hemodynamic forces provide signals to arterial vessels to start recruiting vMCs and complete arterial maturation (Figure 7A).

It has been recently shown that the role of Notch in development goes beyond cell-cell interactions (LaFoya et al., 2016). Recent data in zebrafish suggest the existence of developmental windows, in which Notch signaling can exert its functions during vascular development (Kim et al., 2014, Quillien et al., 2014). It is also conceivable that this dynamic requirement of Notch has different functions during zebrafish vascular development. Before the onset of circulation, Notch that is activated by Shh-VEGF signaling controls artery and vein differentiation (Lawson and Weinstein, 2002). When blood flow reaches a magnitude that is able to activate shear-stress-dependent mechanisms, Notch leads to vMC recruitment and differentiation (Figure 7B).

The role of shear stress in Notch activation has been previously reported but never clearly understood (Jahnsen et al., 2015, Masumura et al., 2009, Obi et al., 2009). We suggest that primary cilia are responsible for the flow-dependent Notch activation that leads to vascular myogenesis. Endothelial primary cilia are microtubule-based sensory organelles protruding into the lumen of blood vessels. Strong attention has recently been given to primary cilia as a possible shear stress sensor in vascular endothelial cells (Ando and Yamamoto, 2013). Recently, Goetz et al. found that zebrafish EC cilia are highly sensitive to shear stress forces from 24 hpf onward (Goetz et al., 2014). We find that endothelial cilia have a critical role in vascular maturation by transducing low-magnitude mechanical stimuli into the molecular signal. Mechanistically, cilia bending after shear stress might function as a lever that alters the cytoskeletal structure, leading to changes in defined biological events (e.g., endocytosis) that alter activation of various receptors, including Notch (Shergill et al., 2012). Not surprisingly, a role for the primary cilium in activating Notch signaling has been previously reported during epidermal differentiation and skin development, perhaps indicating a conserved mechanism of action of cilia on Notch signaling in different species and tissues (Ezratty et al., 2011).

Genetic studies in mice have shown *Foxc1* and *Foxc2* have important functions in arterial endothelial specification as critical downstream effectors of Notch signaling (Hayashi and Kume, 2008, Seo et al., 2006). In zebrafish, *foxc1a* and *foxc1b* (orthologs of mammalian *Foxc1* and *Foxc2*, respectively) are expressed in the developing vasculature, with *foxc1a* expressed in both arteries and veins, whereas *foxc1b* is detected mainly in arteries (Jang et al., 2015, Skarie and Link, 2009). Inactivation of these genes does not change artery or vein differentiation; however, it has been reported that both *foxc1a* and *foxc1b* are required in early hemogenic endothelium for definitive hematopoiesis (Jang et al., 2015). Interestingly, *foxc1a* and *foxc1b* expression remains endothelial restricted after definitive hematopoiesis, supporting an additional vascular role for the FoxC family later during development. In agreement with this observation, we discovered that *foxc1b* (but not *foxc1a*) expression is indeed necessary and sufficient to drive vMC recruitment and differentiation during zebrafish cardiovascular development. Our data indicate that Foxc1b promotes vascular myogenesis and that its function during arterial maturation is flow and Notch dependent. Although different combinations of endothelial genes may be direct transcriptional targets of these FoxC proteins (De Val and Black, 2009), a recent report supports the idea that *foxc1* expression regulates *Pdgf* signaling, which has a central role in the development of the vascular smooth muscle cell lineage, including pericytes and mesangial cells (French et al., 2014). Although the signals released or expressed by *foxc1b* in endothelial cells that are required for vMC recruitment and differentiation need to be identified, our data indicate that shear stress and cilia-dependent Notch signaling activates a foxc1b-dependent endothelial autonomous program that leads to vascular myogenesis.

This understanding of mural cell development and differentiation may help to decipher the molecular processes underlying vascular disease, such as tumor angiogenesis, and may allow chemical and drug screening for use in vascular-myogenesis-related medical therapies.

## Experimental Procedures

### Zebrafish Lines

Zebrafish were maintained under standard laboratory conditions and protocols. The *Tg(kdrl:egfp)*^*s843*^, *Tg(kdrl:CAAX-mcherry)*^*uto2*^, *Tg(tp1:egfp)*^*um14*^, *Tg(flt1:Mmu.Fos-EGFP)*^*wz2*^, *Tg(−5.2lyve1b:DsRed)*^*nz101*^, *TgBAC(dll4:GAL4FF)*^*hu10049*^, and *Tg(5xUAS-E1b:6xMYC-notch1a)*^*kca3*^*, Tg(gata1a:DsRed)*^*sd2*^ lines have been described previously (Hermkens et al., 2015, Mugoni et al., 2013, Nicenboim et al., 2015, Okuda et al., 2012, Quillien et al., 2014, Scheer and Campos-Ortega, 1999, Traver et al., 2003). Generation of the *Tg(acta2:mcherry)*^*uto5*^, *Tg(tagln:CAAX-egfp)*^*uto37*^ lines is described in the Supplemental Information. Following fertilization, eggs were collected, and embryos were grown in the presence of 0.003% 1-phenyl-2-thiourea (PTU) (Sigma-Aldrich) to prevent the formation of melanin pigment.

### vMC Coverage Quantification

Quantification of vMC-specific fluorescence intensity on *Tg(acta2:mcherry)*^*uto5*^ was performed using ImageJ software (v1.44 public domain software, https://imagej.nih.gov/ij/). Z-projections were used to measure fluorescence intensity, which was defined as the following: CTCF = integrated density − (area selected × mean fluorescence of background). Fluorescence intensity in treated embryos was normalized to the intensity in controls. Colocalization analyses were performed with the Coloc tools of Imaris software (Bitplane) and ImageJ (colocalization test plugin) and were performed on 15 z-stacks of 10 images each.

### Integratable CRISPR Vector for Endothelial-Specific Gene Targeting and T7E1 Assay

The method has been described previously (Ablain et al., 2015). Briefly, we generated an integratable CRISPR vector for endothelial-specific gene targeting by introducing the *fli* promoter into the pDestTol2pA2-U6:gRNA (guided RNA) (Addgene #63157) by gateway cloning. Verified gRNA target oligos of *ift88*, *foxc1a*, and *foxc1b* were designed as indicated in Figures S1–S13 and inserted into the above plasmid to make the final injection constructs called fli-*ift88*cKO, fli-*foxc1acKO*, and lli-*foxc1b* conditional knock-out (cKO). These plasmids were injected together with Tol2 mRNA into one- to two-cell-stage embryos, and ten GFP-positive or -negative embryos at 2–4 dpf stages were lysated for genomic DNA extraction and analyzed in a T7E1 assay as described (Ablain et al., 2015, Kim et al., 2009). Briefly, the target site flanking sequence was amplified by PCR using primers in Figures S1–S13. The 200-ng PCR product was mixed with 2 μL of NEBuffer 2 in a total of 19 μL of volume to run a hybridization reaction in a thermocycler: 5 min, 95°C; ramped down to 85°C at a rate of −2°C/s; and then ramped down to 25°C at a rate of −1°C/s. Then 1 μL of T7E1 enzyme (NEB) was added and incubated at 37°C for 15 min. Finally, the samples were loaded in 2% agarose gel.

See also the Supplemental Experimental Procedures.

## Author Contributions

D.G. characterized the vMCs and their flow-dependent recruitment during zebrafish development. X.C. developed the endothelial-specific CRISPR/Cas9 technology and performed the foxc1b and ift88 experiments. C.M. performed the LSS and CBD experiments. All authors planned and discussed the entire project together. M.M.S. coordinated the work and wrote the manuscript.

## Supplemental Information

Document S1. Supplemental Experimental Methods and Figures S1–S13

Movie S1. DII4 Inhibition Alters Blood Flow, in Support of Data Shown in Figure 4 Spinning disk microscopy shows blood circulation (red) in ctrl (A) and *dll4* KD (B) in *Tg(tp1:egfp)*^*um14*^*Tg(gata1:DsRed)*^*sd2*^ at 4 dpf. In *dll4* KD, only in functional SeA Notch is active.

Document S2. Article plus Supplemental Information

## Footnotes

Supplemental Information includes Supplemental Experimental Procedures, 13 figures, and one movie and can be found with this article online at http://dx.doi.org/10.1016/j.celrep.2016.12.044.

## References

[bib1] Ablain J., Durand E.M., Yang S., Zhou Y., Zon L.I. (2015). A CRISPR/Cas9 vector system for tissue-specific gene disruption in zebrafish. Dev. Cell.

[bib2] Ando J., Yamamoto K. (2013). Flow detection and calcium signalling in vascular endothelial cells. Cardiovasc. Res..

[bib3] Anton H., Harlepp S., Ramspacher C., Wu D., Monduc F., Bhat S., Liebling M., Paoletti C., Charvin G., Freund J.B. (2013). Pulse propagation by a capacitive mechanism drives embryonic blood flow. Development.

[bib4] Armulik A., Genové G., Betsholtz C. (2011). Pericytes: developmental, physiological, and pathological perspectives, problems, and promises. Dev. Cell.

[bib5] Boselli F., Freund J.B., Vermot J. (2015). Blood flow mechanics in cardiovascular development. Cell. Mol. Life Sci..

[bib6] Buschmann I., Pries A., Styp-Rekowska B., Hillmeister P., Loufrani L., Henrion D., Shi Y., Duelsner A., Hoefer I., Gatzke N. (2010). Pulsatile shear and Gja5 modulate arterial identity and remodeling events during flow-driven arteriogenesis. Development.

[bib7] Carmeliet P., Jain R.K. (2011). Principles and mechanisms of vessel normalization for cancer and other angiogenic diseases. Nat. Rev. Drug Discov..

[bib8] De Luca E., Zaccaria G.M., Hadhoud M., Rizzo G., Ponzini R., Morbiducci U., Santoro M.M. (2014). ZebraBeat: a flexible platform for the analysis of the cardiac rate in zebrafish embryos. Sci. Rep..

[bib9] De Val S., Black B.L. (2009). Transcriptional control of endothelial cell development. Dev. Cell.

[bib10] Dinsmore C., Reiter J.F. (2016). Endothelial primary cilia inhibit atherosclerosis. EMBO Rep..

[bib11] Ezratty E.J., Stokes N., Chai S., Shah A.S., Williams S.E., Fuchs E. (2011). A role for the primary cilium in Notch signaling and epidermal differentiation during skin development. Cell.

[bib12] Firestone A.J., Weinger J.S., Maldonado M., Barlan K., Langston L.D., O’Donnell M., Gelfand V.I., Kapoor T.M., Chen J.K. (2012). Small-molecule inhibitors of the AAA+ ATPase motor cytoplasmic dynein. Nature.

[bib13] Fortuna V., Pardanaud L., Brunet I., Ola R., Ristori E., Santoro M.M., Nicoli S., Eichmann A. (2015). Vascular mural cells promote noradrenergic differentiation of embryonic sympathetic neurons. Cell Rep..

[bib14] French C.R., Seshadri S., Destefano A.L., Fornage M., Arnold C.R., Gage P.J., Skarie J.M., Dobyns W.B., Millen K.J., Liu T. (2014). Mutation of FOXC1 and PITX2 induces cerebral small-vessel disease. J. Clin. Invest..

[bib15] Gaengel K., Genové G., Armulik A., Betsholtz C. (2009). Endothelial-mural cell signaling in vascular development and angiogenesis. Arterioscler. Thromb. Vasc. Biol..

[bib16] Garcia-Gonzalez M.A., Outeda P., Zhou Q., Zhou F., Menezes L.F., Qian F., Huso D.L., Germino G.G., Piontek K.B., Watnick T. (2010). Pkd1 and Pkd2 are required for normal placental development. PLoS ONE.

[bib17] Georgijevic S., Subramanian Y., Rollins E.L., Starovic-Subota O., Tang A.C., Childs S.J. (2007). Spatiotemporal expression of smooth muscle markers in developing zebrafish gut. Dev. Dyn..

[bib18] Goetz S.C., Anderson K.V. (2010). The primary cilium: a signalling centre during vertebrate development. Nat. Rev. Genet..

[bib19] Goetz J.G., Steed E., Ferreira R.R., Roth S., Ramspacher C., Boselli F., Charvin G., Liebling M., Wyart C., Schwab Y. (2014). Endothelial cilia mediate low flow sensing during zebrafish vascular development. Cell Rep..

[bib20] Hahn C., Schwartz M.A. (2009). Mechanotransduction in vascular physiology and atherogenesis. Nat. Rev. Mol. Cell Biol..

[bib21] Hayashi H., Kume T. (2008). Foxc transcription factors directly regulate Dll4 and Hey2 expression by interacting with the VEGF-Notch signaling pathways in endothelial cells. PLoS ONE.

[bib22] Herbert S.P., Stainier D.Y. (2011). Molecular control of endothelial cell behaviour during blood vessel morphogenesis. Nat. Rev. Mol. Cell Biol..

[bib23] Hermkens D.M., van Impel A., Urasaki A., Bussmann J., Duckers H.J., Schulte-Merker S. (2015). Sox7 controls arterial specification in conjunction with hey2 and efnb2 function. Development.

[bib24] Hierck B.P., Van der Heiden K., Alkemade F.E., Van de Pas S., Van Thienen J.V., Groenendijk B.C., Bax W.H., Van der Laarse A., Deruiter M.C., Horrevoets A.J. (2008). Primary cilia sensitize endothelial cells for fluid shear stress. Dev. Dyn..

[bib25] Jahnsen E.D., Trindade A., Zaun H.C., Lehoux S., Duarte A., Jones E.A. (2015). Notch1 is pan-endothelial at the onset of flow and regulated by flow. PLoS ONE.

[bib26] Jang I.H., Lu Y.F., Zhao L., Wenzel P.L., Kume T., Datta S.M., Arora N., Guiu J., Lagha M., Kim P.G. (2015). Notch1 acts via Foxc2 to promote definitive hematopoiesis via effects on hemogenic endothelium. Blood.

[bib27] Johnson J.L. (2014). Emerging regulators of vascular smooth muscle cell function in the development and progression of atherosclerosis. Cardiovasc. Res..

[bib28] Kallakuri S., Yu J.A., Li J., Li Y., Weinstein B.M., Nicoli S., Sun Z. (2015). Endothelial cilia are essential for developmental vascular integrity in zebrafish. J. Am. Soc. Nephrol..

[bib29] Kim H.J., Lee H.J., Kim H., Cho S.W., Kim J.S. (2009). Targeted genome editing in human cells with zinc finger nucleases constructed via modular assembly. Genome Res..

[bib30] Kim A.D., Melick C.H., Clements W.K., Stachura D.L., Distel M., Panáková D., MacRae C., Mork L.A., Crump J.G., Traver D. (2014). Discrete Notch signaling requirements in the specification of hematopoietic stem cells. EMBO J..

[bib31] Kramer-Zucker A.G., Olale F., Haycraft C.J., Yoder B.K., Schier A.F., Drummond I.A. (2005). Cilia-driven fluid flow in the zebrafish pronephros, brain and Kupffer’s vesicle is required for normal organogenesis. Development.

[bib32] LaFoya B., Munroe J.A., Mia M.M., Detweiler M.A., Crow J.J., Wood T., Roth S., Sharma B., Albig A.R. (2016). Notch: A multi-functional integrating system of microenvironmental signals. Dev. Biol..

[bib33] Lawson N.D., Weinstein B.M. (2002). Arteries and veins: making a difference with zebrafish. Nat. Rev. Genet..

[bib34] Lawson N.D., Scheer N., Pham V.N., Kim C.H., Chitnis A.B., Campos-Ortega J.A., Weinstein B.M. (2001). Notch signaling is required for arterial-venous differentiation during embryonic vascular development. Development.

[bib35] Lawson N.D., Vogel A.M., Weinstein B.M. (2002). Sonic hedgehog and vascular endothelial growth factor act upstream of the Notch pathway during arterial endothelial differentiation. Dev. Cell.

[bib36] le Noble F., Moyon D., Pardanaud L., Yuan L., Djonov V., Matthijsen R., Bréant C., Fleury V., Eichmann A. (2004). Flow regulates arterial-venous differentiation in the chick embryo yolk sac. Development.

[bib37] Majesky M.W. (2007). Developmental basis of vascular smooth muscle diversity. Arterioscler. Thromb. Vasc. Biol..

[bib38] Masumura T., Yamamoto K., Shimizu N., Obi S., Ando J. (2009). Shear stress increases expression of the arterial endothelial marker ephrinB2 in murine ES cells via the VEGF-Notch signaling pathways. Arterioscler. Thromb. Vasc. Biol..

[bib39] Mugoni V., Postel R., Catanzaro V., De Luca E., Turco E., Digilio G., Silengo L., Murphy M.P., Medana C., Stainier D.Y. (2013). Ubiad1 is an antioxidant enzyme that regulates eNOS activity by CoQ10 synthesis. Cell.

[bib40] Nicenboim J., Malkinson G., Lupo T., Asaf L., Sela Y., Mayseless O., Gibbs-Bar L., Senderovich N., Hashimshony T., Shin M. (2015). Lymphatic vessels arise from specialized angioblasts within a venous niche. Nature.

[bib41] Nicoli S., Standley C., Walker P., Hurlstone A., Fogarty K.E., Lawson N.D. (2010). MicroRNA-mediated integration of haemodynamics and Vegf signalling during angiogenesis. Nature.

[bib42] Obi S., Yamamoto K., Shimizu N., Kumagaya S., Masumura T., Sokabe T., Asahara T., Ando J. (2009). Fluid shear stress induces arterial differentiation of endothelial progenitor cells. J. Appl. Physiol. (1985).

[bib43] Okuda K.S., Astin J.W., Misa J.P., Flores M.V., Crosier K.E., Crosier P.S. (2012). lyve1 expression reveals novel lymphatic vessels and new mechanisms for lymphatic vessel development in zebrafish. Development.

[bib44] Owens G.K., Kumar M.S., Wamhoff B.R. (2004). Molecular regulation of vascular smooth muscle cell differentiation in development and disease. Physiol. Rev..

[bib45] Pfaltzgraff E.R., Bader D.M. (2015). Heterogeneity in vascular smooth muscle cell embryonic origin in relation to adult structure, physiology, and disease. Dev. Dyn..

[bib46] Quillien A., Moore J.C., Shin M., Siekmann A.F., Smith T., Pan L., Moens C.B., Parsons M.J., Lawson N.D. (2014). Distinct Notch signaling outputs pattern the developing arterial system. Development.

[bib47] Samsa L.A., Givens C., Tzima E., Stainier D.Y., Qian L., Liu J. (2015). Cardiac contraction activates endocardial Notch signaling to modulate chamber maturation in zebrafish. Development.

[bib48] Santoro M.M., Pesce G., Stainier D.Y. (2009). Characterization of vascular mural cells during zebrafish development. Mech. Dev..

[bib49] Scheer N., Campos-Ortega J.A. (1999). Use of the Gal4-UAS technique for targeted gene expression in the zebrafish. Mech. Dev..

[bib50] Seo S., Fujita H., Nakano A., Kang M., Duarte A., Kume T. (2006). The forkhead transcription factors, Foxc1 and Foxc2, are required for arterial specification and lymphatic sprouting during vascular development. Dev. Biol..

[bib51] Shergill B., Meloty-Kapella L., Musse A.A., Weinmaster G., Botvinick E. (2012). Optical tweezers studies on Notch: single-molecule interaction strength is independent of ligand endocytosis. Dev. Cell.

[bib52] Siekmann A.F., Lawson N.D. (2007). Notch signalling and the regulation of angiogenesis. Cell Adhes. Migr..

[bib53] Siekmann A.F., Lawson N.D. (2007). Notch signalling limits angiogenic cell behaviour in developing zebrafish arteries. Nature.

[bib54] Simons M., Eichmann A. (2015). Molecular controls of arterial morphogenesis. Circ. Res..

[bib55] Skarie J.M., Link B.A. (2009). FoxC1 is essential for vascular basement membrane integrity and hyaloid vessel morphogenesis. Invest. Ophthalmol. Vis. Sci..

[bib56] Swift M.R., Weinstein B.M. (2009). Arterial-venous specification during development. Circ. Res..

[bib57] Traver D., Paw B.H., Poss K.D., Penberthy W.T., Lin S., Zon L.I. (2003). Transplantation and in vivo imaging of multilineage engraftment in zebrafish bloodless mutants. Nat. Immunol..

[bib58] Tsujikawa, M., and Malicki, J. (2004). Intraflagellar transport genes are essential for differentiation and survival of vertebrate sensory neurons. *42*, 703–716. 10.1016/s0896-6273(04)00268-5 15182712

[bib59] Udan R.S., Vadakkan T.J., Dickinson M.E. (2013). Dynamic responses of endothelial cells to changes in blood flow during vascular remodeling of the mouse yolk sac. Development.

[bib60] van Dijk C.G., Nieuweboer F.E., Pei J.Y., Xu Y.J., Burgisser P., van Mulligen E., el Azzouzi H., Duncker D.J., Verhaar M.C., Cheng C. (2015). The complex mural cell: pericyte function in health and disease. Int. J. Cardiol..

[bib61] van Impel A., Zhao Z., Hermkens D.M., Roukens M.G., Fischer J.C., Peterson-Maduro J., Duckers H., Ober E.A., Ingham P.W., Schulte-Merker S. (2014). Divergence of zebrafish and mouse lymphatic cell fate specification pathways. Development.

[bib62] Wang Y., Pan L., Moens C.B., Appel B. (2014). Notch3 establishes brain vascular integrity by regulating pericyte number. Development.

[bib63] Wang G., Jacquet L., Karamariti E., Xu Q. (2015). Origin and differentiation of vascular smooth muscle cells. J. Physiol..

[bib64] Whitesell T.R., Kennedy R.M., Carter A.D., Rollins E.L., Georgijevic S., Santoro M.M., Childs S.J. (2014). An α-smooth muscle actin (acta2/αsma) zebrafish transgenic line marking vascular mural cells and visceral smooth muscle cells. PLoS ONE.

[bib65] Yoshida T., Owens G.K. (2005). Molecular determinants of vascular smooth muscle cell diversity. Circ. Res..

[bib66] Zhong T.P., Childs S., Leu J.P., Fishman M.C. (2001). Gridlock signalling pathway fashions the first embryonic artery. Nature.

