# Supplementary material for: Cilia Control Vascular Mural Cell Recruitment in Vertebrates
Source: Cell Rep. 2017 Jan 24;18(4):1033–47. doi: 10.1016/j.celrep.2016.12.044 (PMC5289940; doi:10.1016/j.celrep.2016.12.044)
Supplement: Document S1. Supplemental Experimental Methods and Figures S1–S13 [file mmc1.pdf]

Cell Reports, Volume *18*

Supplemental Information

Cilia Control Vascular Mural Cell Recruitment in Vertebrates

Xiaowen Chen, Dafne Gays, Carlo Milia, and Massimo M. Santoro

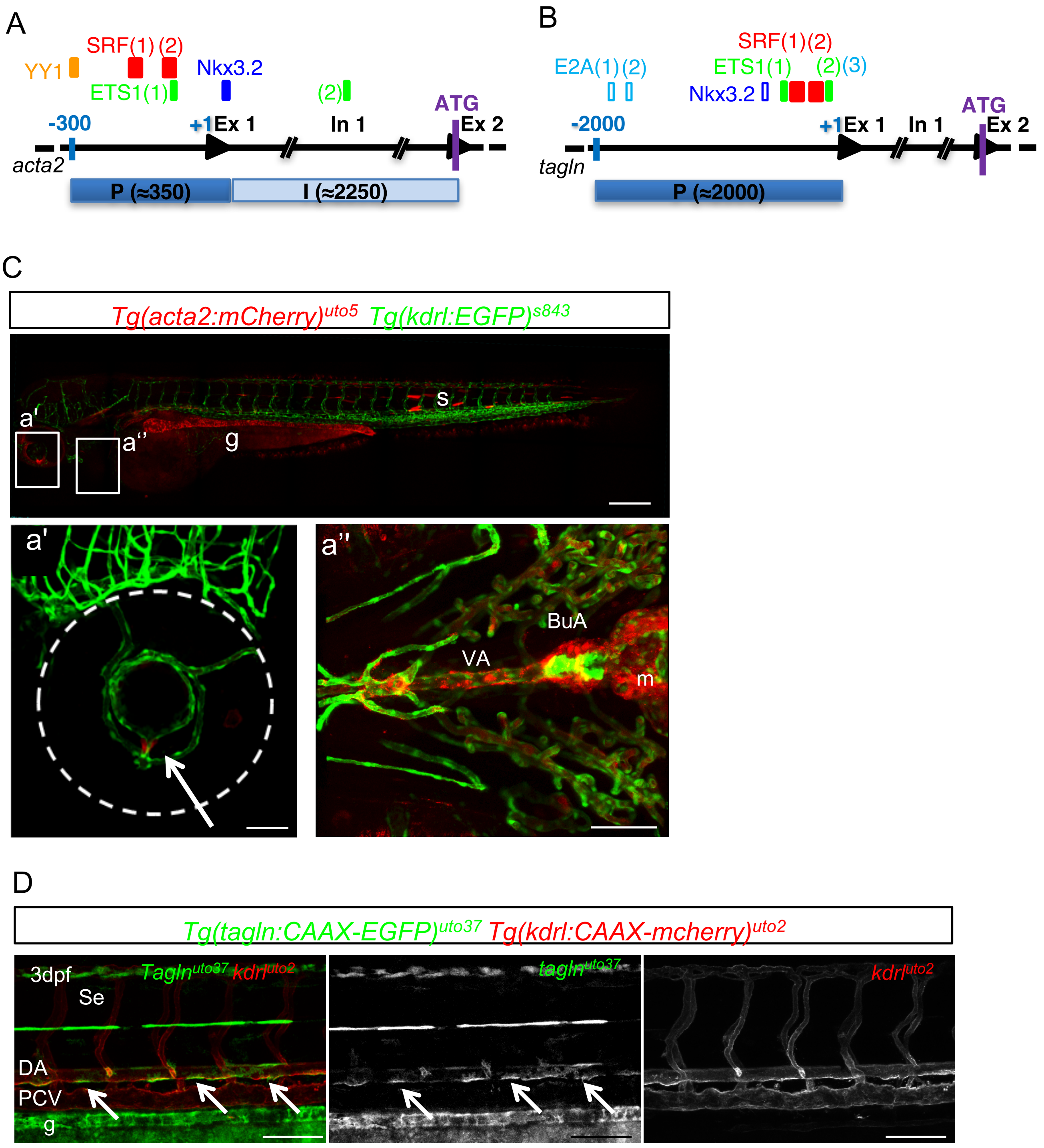

Suppl. Figure 1

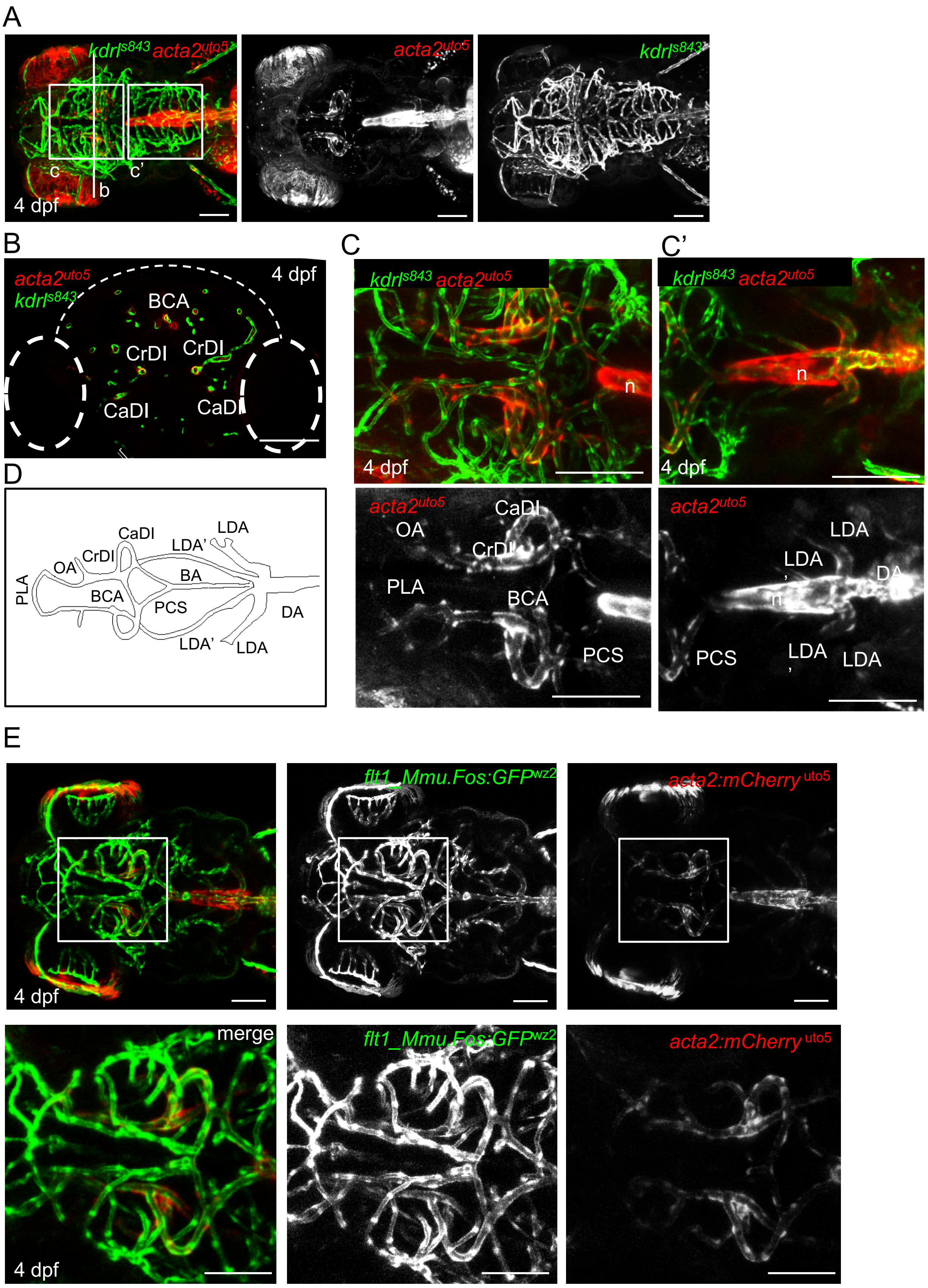

Suppl. Figure 2

A

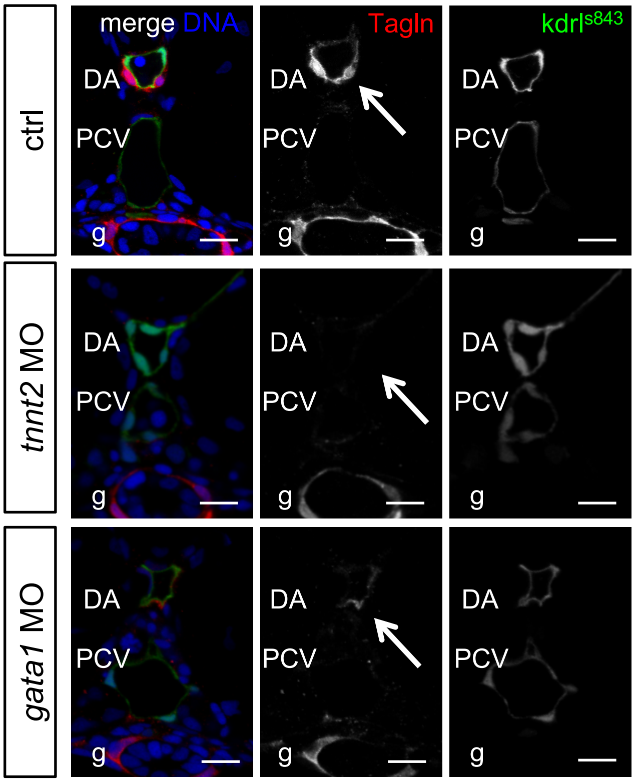

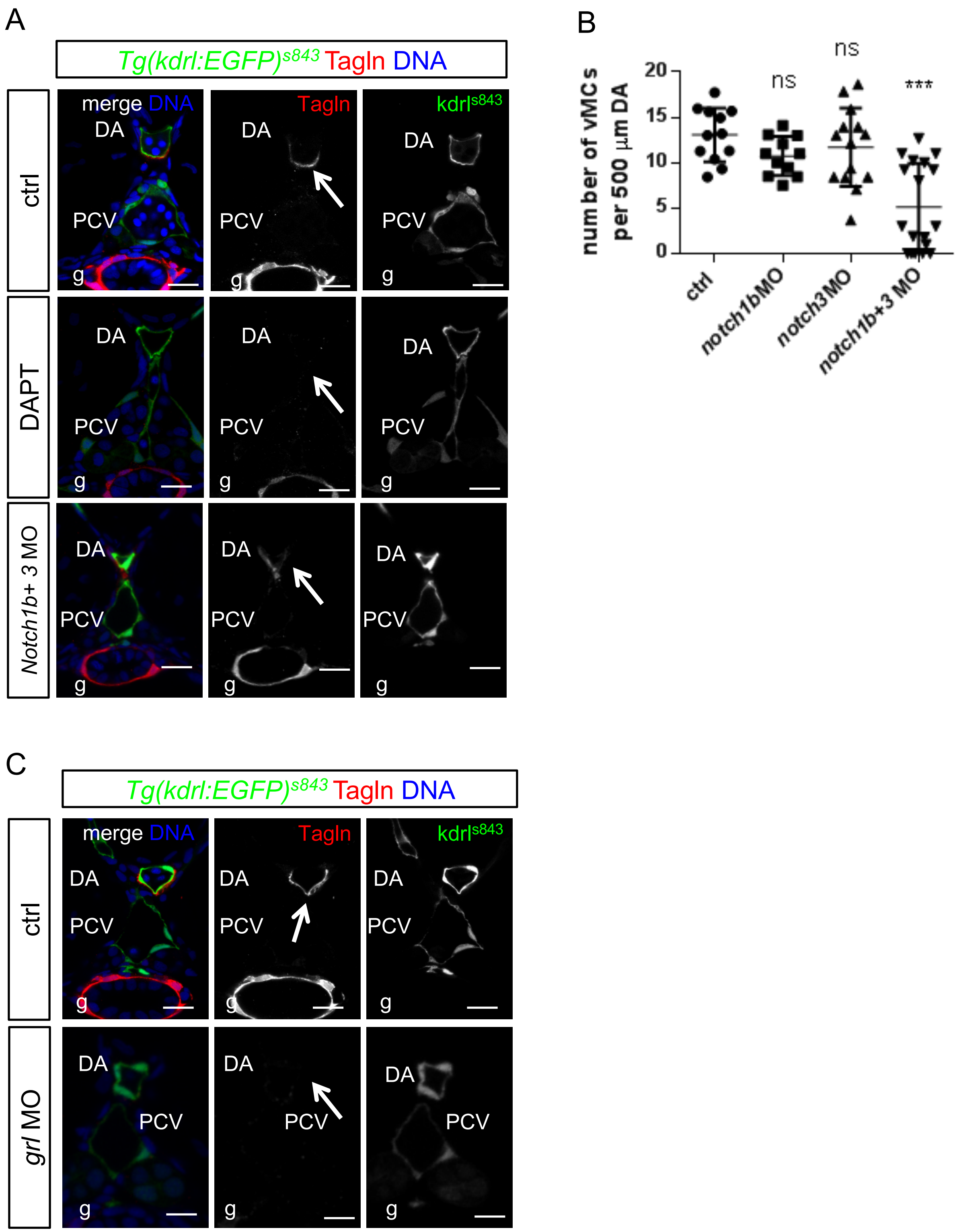

Suppl. Figure 4

A

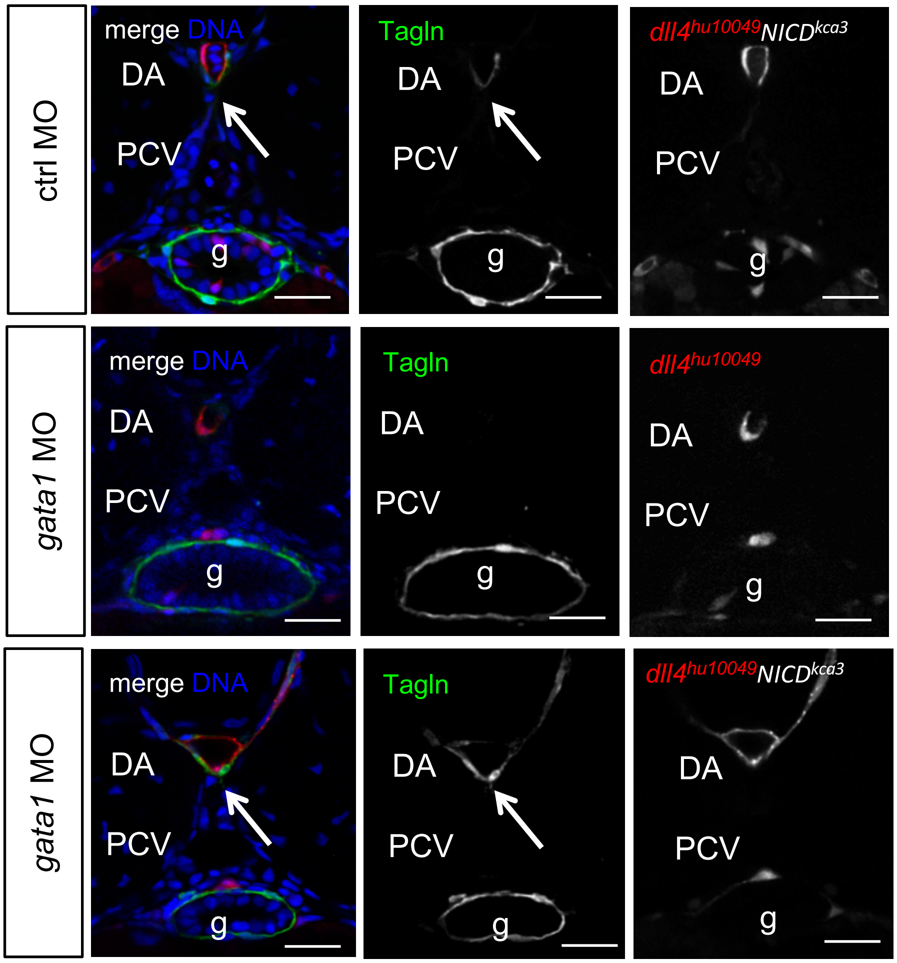

Suppl. Figure 5

A

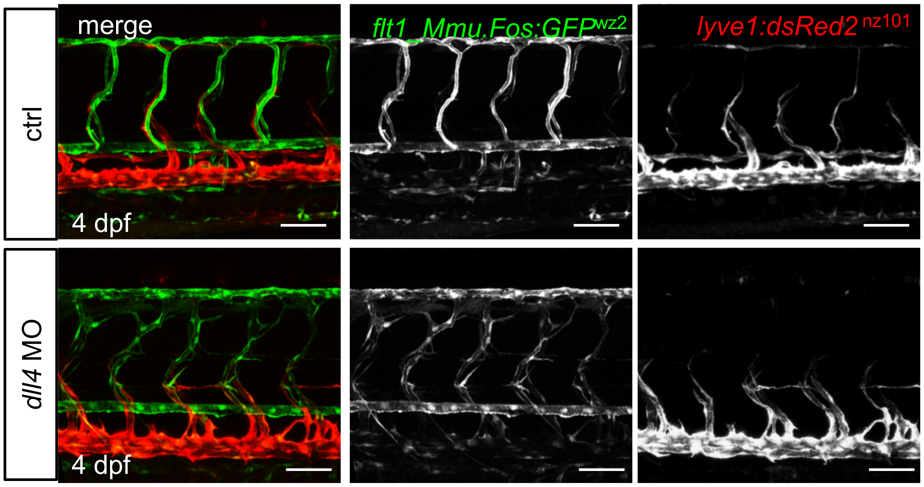

Suppl. Figure 6

A

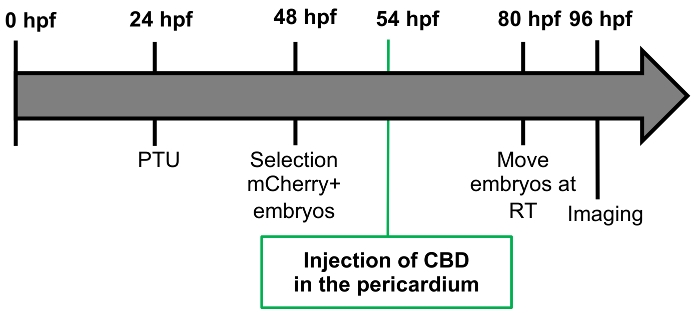

B

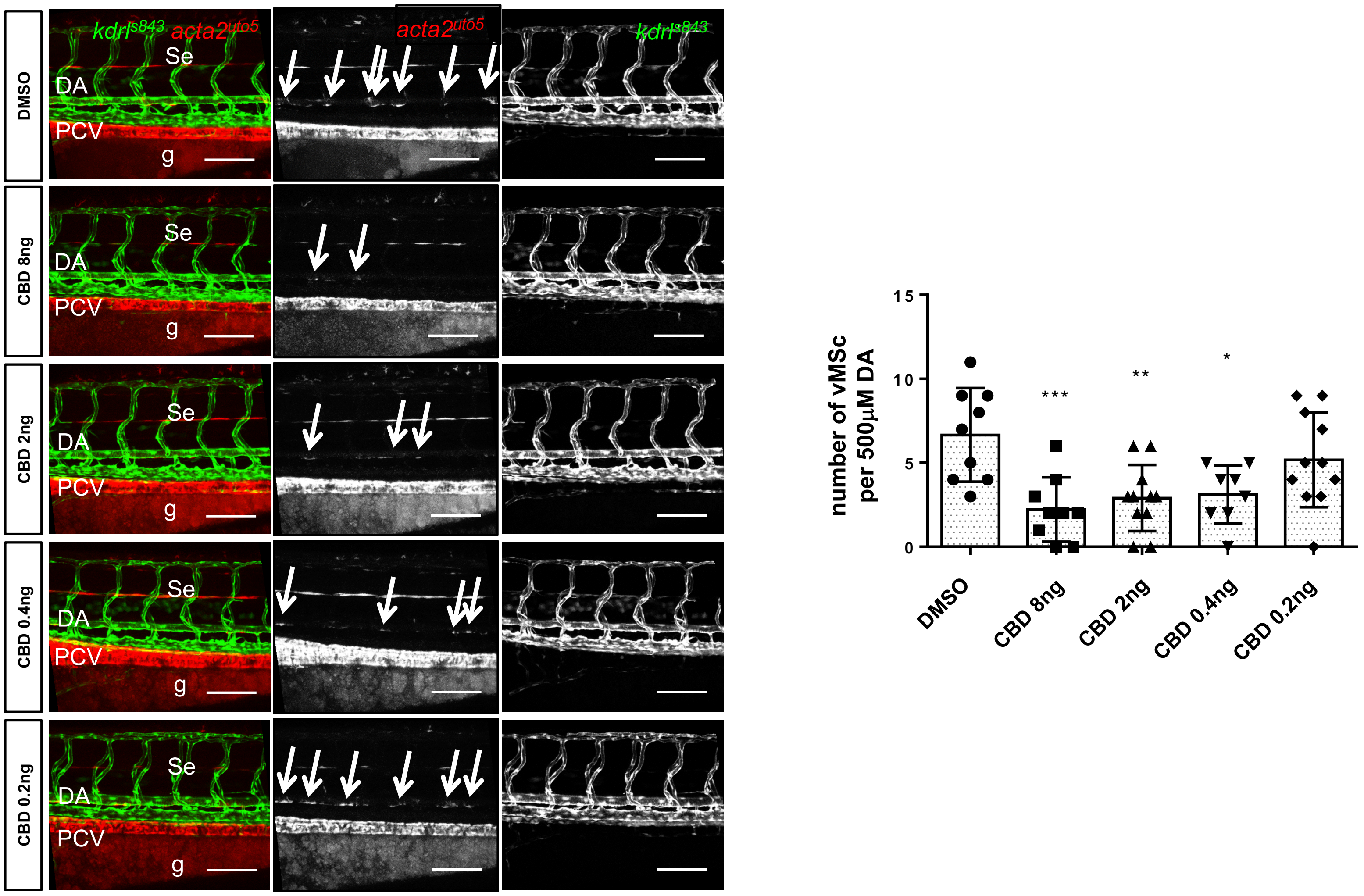

Suppl. Figure 7

A

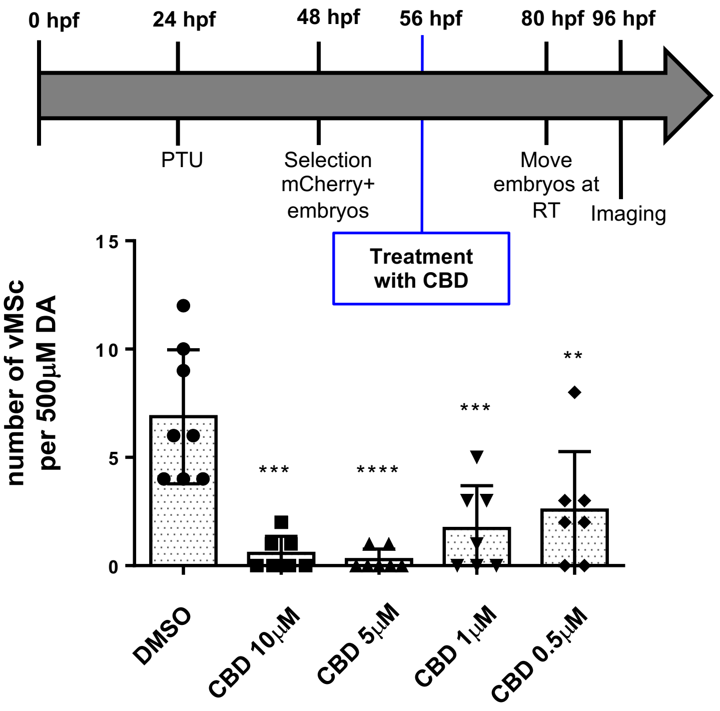

B

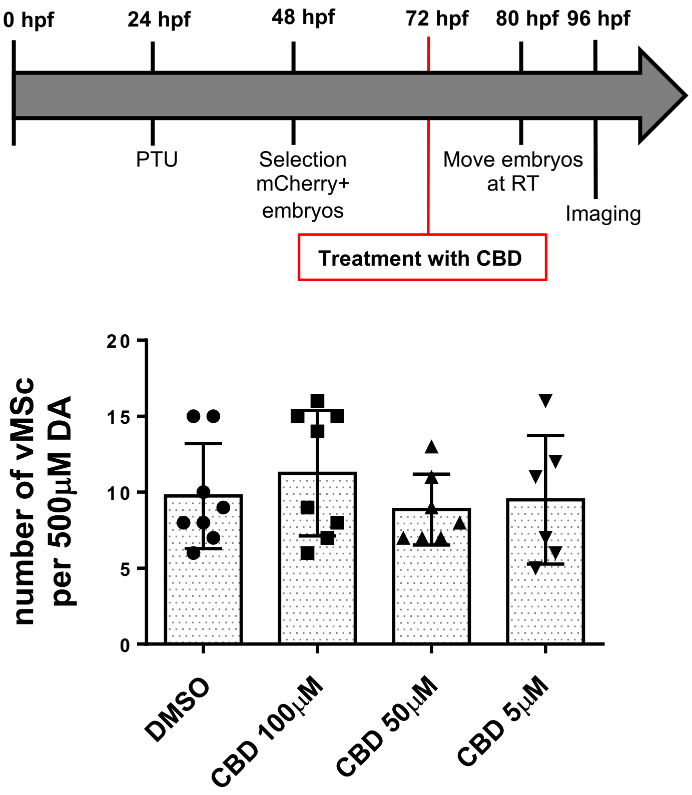

Suppl. Figure 8

A

gRNA target sequences (red indicates NGG region)

foxc1a gRNA target	5'-GGGTGGAAGGGATGCTGTGCGG-3'
foxc1b gRNA target	5'-GGCGGCATGGCTCGCGCATAGG-3'
ift88 gRNA target	5'-GGCTGACCGCTATGCAGAGCTGG-3'

B

p*cmlc2*:GFP;U6:gRNA;*fli*:Cas9 integratable vectors

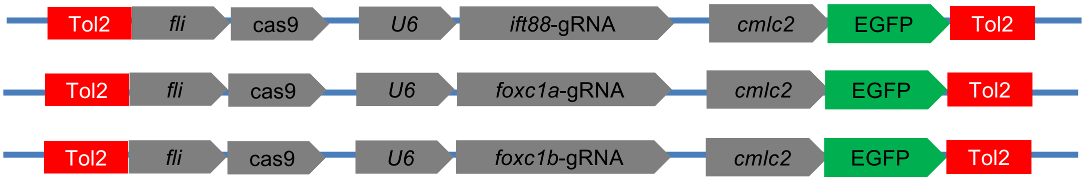

C

Workflow for endothelial cell-specific CRISP-based knockout technology

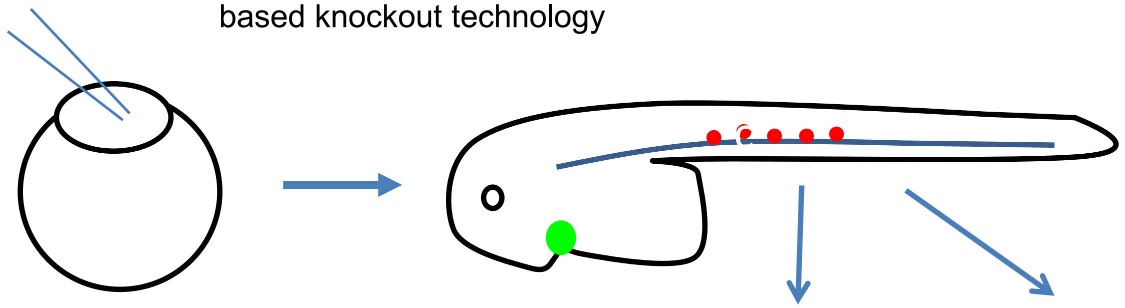

Injection of p*cmlc2*:GFP;U6:gRNA;*fli*:Cas9 intergratable vectors with transposase mRNA into *Tg(acta2:mCherry)*^{uto5} lines.

Analyze vMCs around DA of the *cmlc2*:GFP positive embryos at 80hpf.

Genomic DNA extraction for genotyping using T7E1 assay *as control cmcl2:GFP negative embryso were used

D

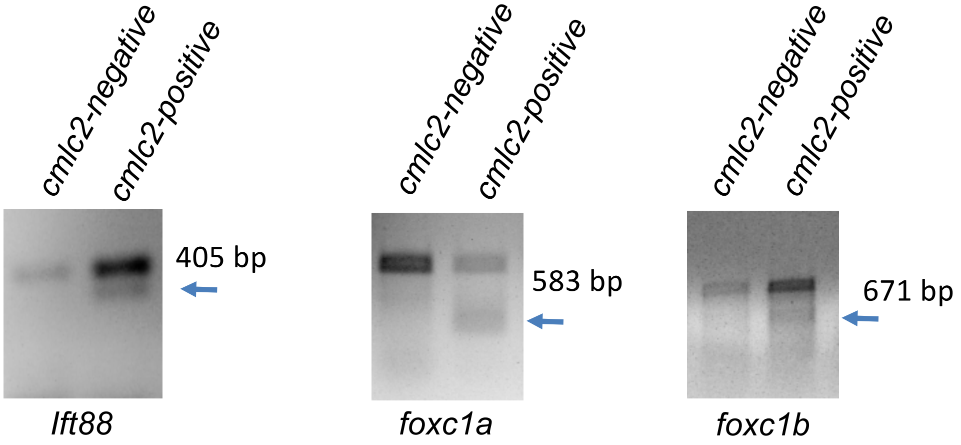

Suppl. Figure 9

E

Primers used for CRISPR plasmid construction

foxc1a-gRNA-For	5'-GGAGGACCGAGGTGTTAAAGgt-3'
foxc1a-gRNA-Rev	5'-CTTTAACACCTCGGTCCTCCga-3'
foxc1b-gRNA-For	5'-GGCGGCATGGCTCGCGCATAgt-3'
foxc1b-gRNA-Rev	5'-TATGCGCGAGCCATGCCGCCga-3'
ift88-gRNA-For	5'-GGCTGACCGCTATGCAGAGCgt-3'
ift88-gRNA-Rev	5'-GCTCTGCATAGCGGTCAGCCga-3'

F

Primers used for T7E1 assay

foxc1a-PCR-For	5'-CGGAAAAGGCAGCTACTGGA-3'
foxc1a-PCR-Rev	5'-GGTAGTCGAGATAGAGTACTGG-3'
foxc1b-PCR-For	5'-CCCTTTAGGCGTTGTGCCTTAT-3'
foxc1b-PCR-Rev	5'-TGTCTGGGCTCTCCGTTTTA-3'
ift88-PCR-For	5'-GTAATGGCTGCCAGTTTAC-3'
ift88-PCR-Rev	5'-TACGTCAAACCTGCATGATG-3'

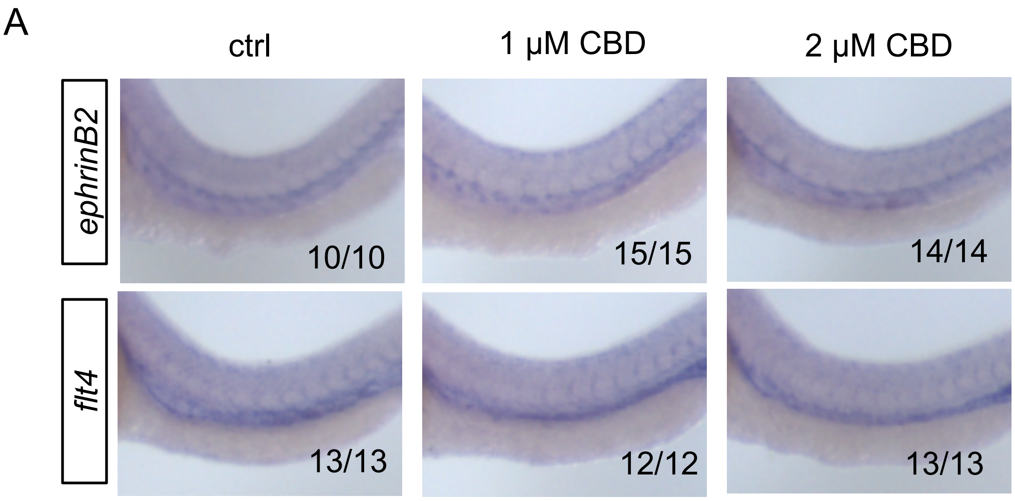

Suppl. Figure 10

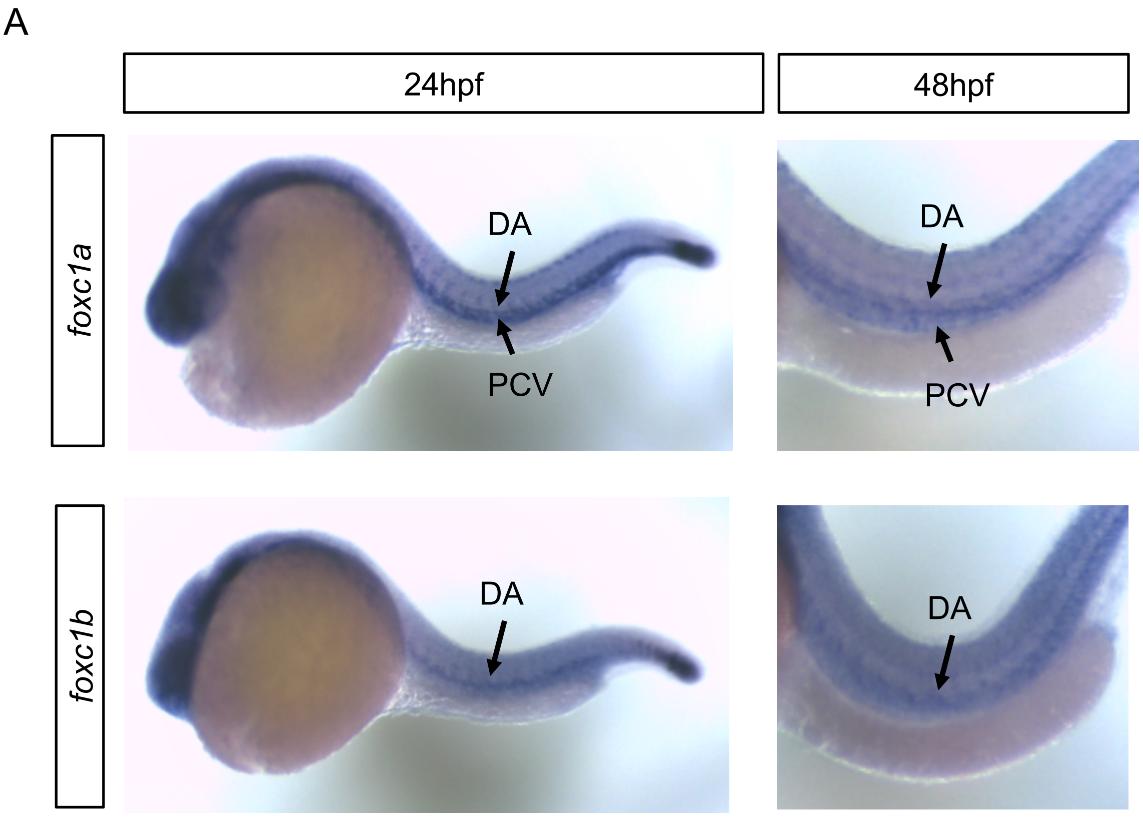
A
24hpf
48hpf
foxc1a
foxc1b
DA
PCV
DA
PCV
DA
DA

A

B

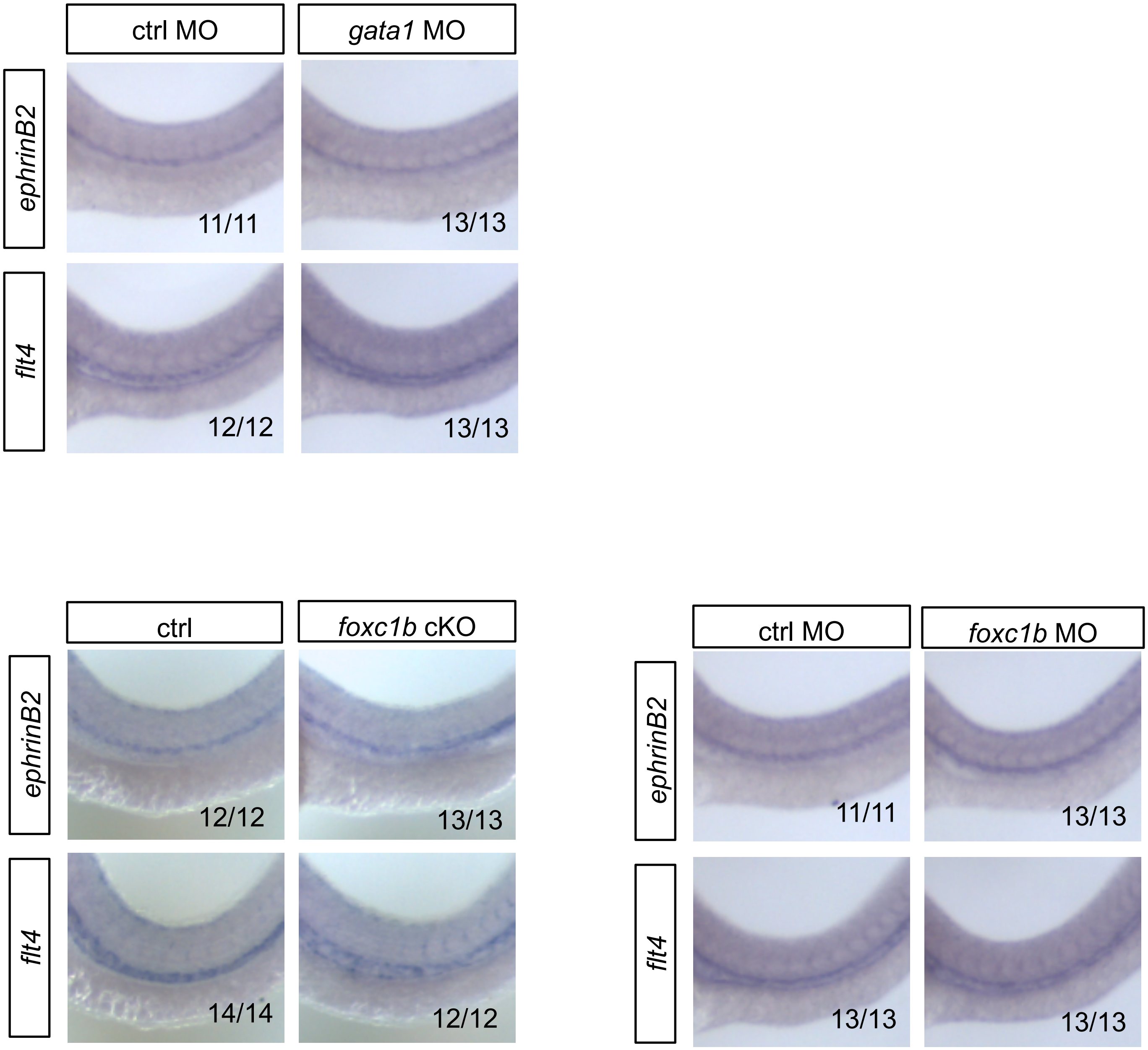

Suppl. Figure 12

A

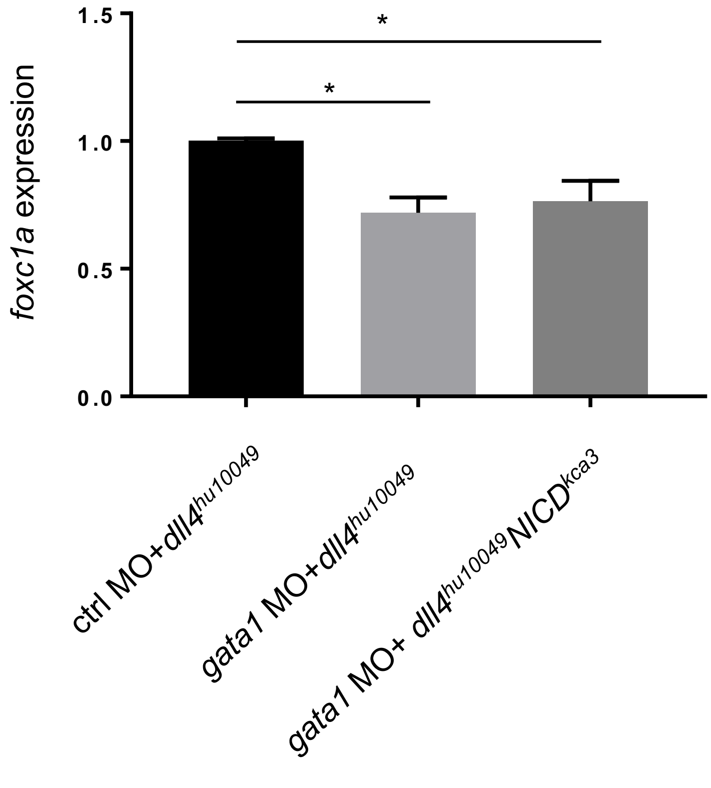

Suppl. Figure 13
