# Supplementary figures and images for: Cilia Control Vascular Mural Cell Recruitment in Vertebrates
Source: Cell Rep. 2017 Jan 24;18(4):1033–47. doi: 10.1016/j.celrep.2016.12.044 (PMC5289940; doi:10.1016/j.celrep.2016.12.044)

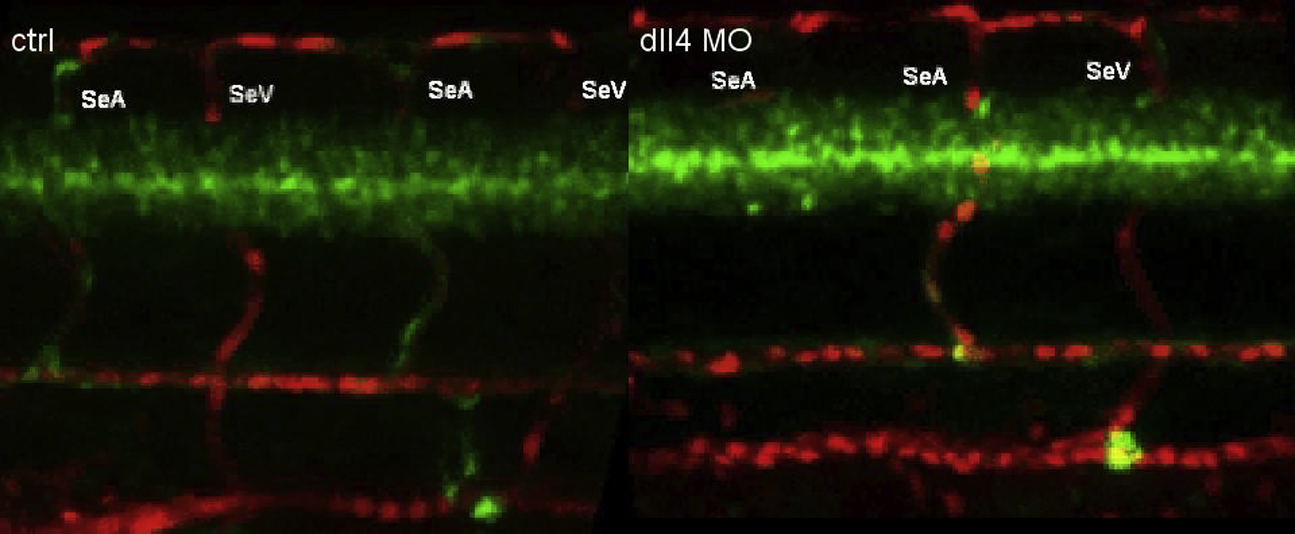

Supplement: Movie S1. DII4 Inhibition Alters Blood Flow, in Support of Data Shown in Figure 4 — Spinning disk microscopy shows blood circulation (red) in ctrl (A) and dll4 KD (B) in Tg(tp1:egfp)um14Tg(gata1:DsRed)sd2 at 4 dpf. In dll4 KD, only in functional SeA Notch is active. [file mmc2.jpg]
